# First-principles calculations to investigate structural, electronic, optical, elastic, mechanical and phonon properties of novel Q_3_GaBr_6_ (Q = Na and K) for next-generation lead-free solar cells

**DOI:** 10.1039/d5ra10011a

**Published:** 2026-02-06

**Authors:** Rifat Rafiu, Md. Sakib Hasan, Md. Azizur Rahman, Imtiaz Ahamed Apon, Karim Kriaa, Mohamed Benghanem, S. AlFaify, Noureddine Elboughdiri

**Affiliations:** a Department of Material Science and Engineering, Khulna University of Engineering & Technology (KUET) Khulna-9203 Bangladesh; b Innovative Solar and Energy Materials Laboratory (ISEML), Department of Electrical and Electronic Engineering, Begum Rokeya University Rangpur 5400 Bangladesh azizurrahmanatik49@gmail.com; c Department of Electrical and Electronic Engineering, Bangladesh Army University of Science and Technology (BAUST) Saidpur-5311 Bangladesh; d College of Engineering, Imam Mohammad Ibn Saud Islamic University (IMSIU) Riyadh 11432 Saudi Arabia; e Physics Department, Faculty of Science, Islamic University of Madinah Madinah 42351 Saudi Arabia mbenghanem@iu.edu.sa; f Department of Physics, College of Sciences, King Khalid University P.O. Box 960, AlQura'a Abha 61421 Saudi Arabia; g Chemical Engineering Department, College of Engineering, University of Ha'il P.O. Box 2440 81441 Ha'il Saudi Arabia

## Abstract

Lead-free halide perovskites have emerged as promising alternatives to toxic Pb-based photovoltaic absorbers, yet many candidates suffer from poor stability or unfavorable electronic properties. In this work, we present the first comprehensive first-principles and device-level investigation of the novel vacancy-ordered perovskites Q_3_GaBr_6_ (Q = Na, K) to evaluate their potential for next-generation optoelectronic and solar-cell applications. Density functional theory (DFT) calculations confirm that both compounds crystallize in a stable cubic *Fm*3̄*m* phase with negative formation energies, favorable tolerance factors, and strong Ga–Br bonding within rigid octahedral frameworks. Electronic-structure analysis reveals direct band gaps of 1.445 eV (K_3_GaBr_6_) and 1.991 eV (Na_3_GaBr_6_), with Br-4p states dominating the valence band and Ga-/Q-site orbitals contributing to the conduction band. Optical studies show high absorption (>10^4^ cm^−1^ in the visible region), low reflectivity, strong dielectric response, and pronounced UV absorption, indicating suitability for broadband optoelectronics. Mechanical and phonon analyses further confirm mechanical stability, moderate stiffness, and absence of imaginary phonon modes, while AIMD simulations validate excellent thermal robustness at elevated temperatures. Incorporating DFT-extracted parameters into SCAPS-1D device modeling demonstrates promising photovoltaic performance, with efficiency, current density, and fill factor strongly influenced by absorber thickness, defect density, and doping concentration. Under ideal simulated conditions, the device shows a theoretical upper-limit efficiency of 22.21%. The proposed DFT–SCAPS integrated approach provides an efficient and computationally economical route to screen and optimize lead-free perovskite absorbers, significantly reducing experimental trial-and-error while enabling accurate prediction of photovoltaic performance.

## Abstract 2

Lead-free halide perovskites have emerged as promising alternatives to toxic Pb-based photovoltaic absorbers, yet many candidates suffer from poor stability or unfavorable electronic properties.

## Graphical abstract

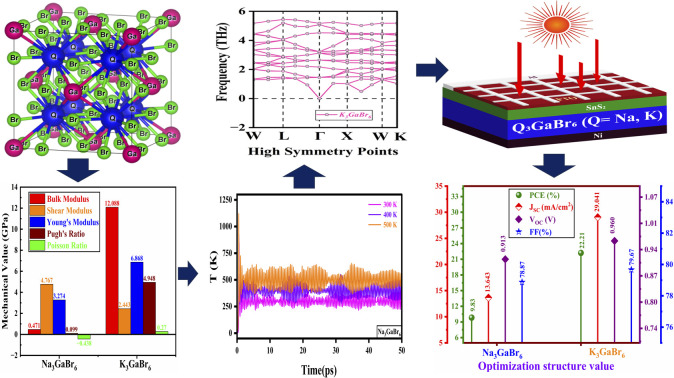

## 1. Introduction

Perovskite solar cells (PSCs) have rapidly emerged as a transformative photovoltaic technology, with power conversion efficiencies (PCEs) rising from 3.8% in 2009 to over 25% for single-junction devices by 2023, while perovskite-silicon tandem cells have reached 29.8%.^1^ The remarkable progress of halide perovskite-based optoelectronic devices represents a major turning point in materials science, primarily driven by their outstanding structural flexibility and exceptional optoelectronic properties.^2–4^ Such rapid advancement is attributed to features such as high optical absorption coefficients, direct and tunable band gaps, long carrier lifetimes, and high defect tolerance. In addition to experimental developments, recent comprehensive reviews have highlighted how combined computational approaches—such as density functional theory (DFT), machine learning, and high-throughput screening—are increasingly essential for guiding the discovery and optimization of lead-free perovskite materials with competitive performance metrics.^5^ However, most high-performing candidates rely on toxic Pb, and additionally, many of these compounds suffer from intrinsic instability when exposed to ambient conditions.^6,7^ The combined issues of toxicity and instability have created an urgent need to identify Pb-free, stable halide perovskites with competitive optoelectronic behavior.

One intuitive approach is to substitute Pb with elements possessing similar electronic configurations, such as tin (Sn^2+^) and germanium (Ge^2+^), both of which belong to the same group as Pb.^8–10^ While these substitutions hold promise, Sn-based perovskites readily oxidize from Sn^2+^ to Sn^4+^, producing degradation by-products such as SnI_2_, which may be nearly as harmful as Pb itself.^11^ Likewise, Ge-based perovskites suffer from rapid oxidation of Ge^2+^ to Ge^4+^ upon exposure to air and moisture, resulting in severe structural and electronic degradation.^12^ Beyond these divalent cations, Group VA elements such as bismuth (Bi^3+^) and antimony (Sb^3+^) have been explored due to their valence electronic configurations (6s^2^6p^0^ for Bi^3+^ and 5s^2^5p^0^ for Sb^3+^), which resemble that of Pb^2+^.^13^ However, their trivalent nature, along with differences in electronegativity and ionic radii, drives a structural shift away from the traditional ABX_3_ perovskite framework toward more stable, ternary halides of the A_3_MX_6_ type structures.^14–16^ Although these materials offer improved stability and reduced toxicity, they often exhibit less favorable optoelectronic characteristics compared to their Pb-based counterparts. Among these, significant attention has been given to the influence of alkaline metal cations on the electrical conductivity of molten cryolites such as K_3_AlF_6_, Rb_3_AlF_6_, and Cs_3_AlF_6_, where variations in ionic size and bonding environments strongly affect ionic mobility and melt behavior.^17^ Additionally, polymorphism in A_3_MF_6_ compounds (A = Rb, Cs; M = Al, Ga) has been investigated, particularly in crystals grown using mixed-halide fluxes, revealing how subtle chemical substitutions and growth conditions can drive the stabilization of multiple structural phases.^14^ Furthermore, first-principles DFT studies on K_3_GaF_6_ have provided valuable insights into its structural, electronic, and optical characteristics, contributing to a broader understanding of fluoride-based materials and their potential technological relevance.^18^ Cs_3_SbX_6_ (X = F, Cl) was shown to exhibit wide band gaps (up to ∼5.5 eV), stable cubic phases, and negative Gibbs free energies, alongside strong optical absorption and high light yields, suggesting potential in both photovoltaic and scintillation applications.^19^ Similarly, Rb_3_SbX_6_ (X = F, Cl, Br, I) displays a systematic reduction in bandgap from 5.47 eV (F) to 2.85 eV (I), consistent with increasing halide ionic radii, while maintaining excellent thermal stability and ductile mechanical properties.^20^ Sodium all-solid-state batteries (ASSBs) with superionic solid electrolytes such as Na_3_MX_6_ (X = Cl, Br, and I) show strong potential for safe and large-scale energy-storage applications.^21^ Such computational studies not only validate the structural and thermodynamic feasibility of A_3_BX_6_-type compounds but also highlight their potential as environmentally benign, tunable materials for next-generation optoelectronics.

To further enhance photovoltaic performance, researchers have begun to explore rudorffite-type Ag–Bi–I perovskites (Ag_3_BiI_6_, Ag_2_BiI_5_, AgBiI_4_) in tandem configurations with silicon. Through SCAPS-1D simulations, the optimization of absorber thickness, carrier transport layers, and defect densities has demonstrated significant efficiency improvements, with simulated tandem efficiencies reaching above 22% for Ag_3_BiI_6_/Si, far exceeding the single-junction values of 5 to 10%.^22^ These findings illustrate the importance of combining first-principles electronic structure modeling with device-level simulations, providing a multi-scale perspective that can guide the practical design of lead-free perovskite solar cells. Stability issues have motivated the development of advanced fabrication methods, such as dynamic casting (DC) combined with ramped annealing (RA), which improves surface coverage, crystallinity, and film density, thereby enhancing device performance. Using this approach, the PCE of inverted planar Ag_3_BiI_6_ devices increased from 0.07% to 1.08%, demonstrating the effectiveness of processing optimization for Pb-free rudorffites.^23^ These considerations highlight the dual challenge of achieving intrinsic material stability alongside effective film and device engineering, both of which are essential for the development of reliable Pb-free photovoltaic absorbers. To date, the vacancy-ordered halide perovskites Q_3_GaBr_6_ (Q = Na, K) have not been reported in prior theoretical or experimental studies, motivating their investigation as potential lead-free photovoltaic materials.

In this work, we employ first-principles density functional theory (DFT) to systematically examine the structural, electronic, optical, mechanical, dynamical, and thermodynamic properties of Na_3_GaBr_6_ and K_3_GaBr_6_. Particular attention is given to evaluating their stability, band structure, and optical absorption characteristics relevant to solar-energy conversion. The DFT-derived material parameters are subsequently incorporated into SCAPS-1D simulations to explore device-level photovoltaic performance. The device results are intended to provide theoretical performance limits rather than realistic device predictions. Key device parameters, including absorber thickness, defect density, and doping concentration, are varied to assess performance trends and identify optimal operating conditions. By combining atomistic insights with device-scale simulations, this study aims to connect intrinsic material properties with photovoltaic behavior. The results are therefore presented as theoretical performance limits intended to guide future experimental exploration of Ga-based vacancy-ordered halide perovskites as promising Pb-free absorber candidates for stable and sustainable solar-cell technologies.

## 2. Computational method

This section describes the theoretical framework and computational procedures adopted to evaluate the structural, electronic, optical, mechanical, vibrational, and thermodynamic properties of Q_3_GaBr_6_ (Q = Na, K). Density Functional Theory (DFT) is employed because of its proven reliability in predicting ground-state properties of crystalline materials, while SCAPS-1D device simulation is used to bridge atomic-scale material properties with macroscopic photovoltaic performance.^24,25^ The combined approach enables a comprehensive multiscale assessment of these materials for solar-cell applications.^26–28^ Density Functional Theory (DFT) simulations were undertaken using the CASTEP code within the Materials Studio framework^29,30^ to systematically evaluate the structural, electronic, optical, mechanical, photonic, thermodynamic, and charge-related properties of Q_3_GaBr_6_ (Q = Na, K). The exchange–correlation interactions were described by the Generalized Gradient Approximation based on the Perdew–Burke–Ernzerhof (GGA-PBE) functional,^31^ mGGA-RSCAN and HSE06 while a plane-wave basis set with a cutoff energy of 600 eV and Norm-Conserving Pseudopotentials (NCPP) was employed for all elements.^32^ This strategy ensures high computational efficiency by minimizing calculation time while retaining sufficient accuracy for large-scale material screening and device optimization. Brillouin-zone integrations were performed using a 7 × 7 × 7 Monkhorst–Pack *k*-point mesh for structural optimization and SCF convergence,^33^ whereas, a denser 12 × 12 × 12 grid was used during non-SCF calculations to obtain high-resolution electronic band structures and projected density of states.^34^ The total energy convergence criterion was set to 10^−8^ eV per atom, and structural relaxation proceeded until the Hellmann–Feynman forces fell below 0.01 eV Å^−1^ and the residual stresses were under 0.02 GPa.^35^ The optimized structures were employed to determine lattice parameters, bond lengths, formation enthalpy, and unit-cell volume to confirm structural and thermodynamic stability. Subsequent calculations were mainly performed using the GGA-PBE functional because of its computational efficiency and reliable accuracy, while the mGGA-RSCAN functional was used selectively for improved band-gap estimation despite its higher computational cost.^36^ Electronic band structures and DOS/PDOS were calculated to analyze band gaps and orbital contributions, while Mulliken, Hirshfeld, and Bader analyses were used to examine charge transfer and bonding characteristics. Charge density and charge density difference maps were employed to visualize electron distribution and bonding nature. Optical properties were derived from the complex dielectric function to evaluate absorption, refractive index, conductivity, reflectivity, and energy-loss behavior relevant to optoelectronic applications.^37^ Mechanical properties were evaluated from elastic constants obtained by the stress–strain method to determine elastic moduli and confirm mechanical stability. Phonon dispersion curves and phonon density of states were calculated to verify dynamic stability and analyze lattice vibrations. Thermodynamic parameters were derived from phonon data using the quasi-harmonic Debye model.

Additionally, AIMD simulations were carried out at physically realistic temperatures of 300 K, 400 K, and 500 K to represent ambient and moderately elevated operating conditions relevant for halide perovskite solar-cell applications. The simulations were performed in the NVT ensemble using a Nosé–Hoover thermostat with a time step of 1.0 fs and a total simulation time of 8 to 10 ps, following sufficient equilibration. These temperatures were selected to ensure physically meaningful evaluation of thermal stability and structural robustness.

While DFT provides accurate intrinsic material properties, photovoltaic performance ultimately depends on device-level factors such as charge transport, recombination, and interface effects; therefore, to bridge this gap between atomistic predictions and practical device behavior, SCAPS-1D simulations were incorporated to translate the DFT-derived parameters into realistic solar-cell characteristic. The DFT-derived electronic and optical parameters, including bandgap, electron affinity, dielectric constant, and effective carrier masses, were incorporated into the SCAPS model. A systematic variation of absorber thickness (300 to 2100 nm), shallow acceptor density (10^13^ to 10^20^ cm^−3^), and total defect density (10^10^ to 10^17^ cm^−3^) was performed to identify optimal device configurations. Standard operating conditions were applied, including AM1.5G illumination at 1000 mW cm^−2^ and a temperature of 300 K, with appropriate series and shunt resistances and interface defect states introduced at ETL/absorber (Q_3_GaBr_6_) interfaces. The current density–voltage (*J*–*V*) characteristics were simulated to extract key photovoltaic parameters such as power conversion efficiency (PCE), open-circuit voltage (*V*_OC_), short-circuit current density (*J*_SC_), and fill factor (FF). Additionally, quantum efficiency (QE) spectra were generated to evaluate wavelength-dependent carrier generation, absorption, and collection efficiency. This combined DFT–SCAPS framework offers a comprehensive understanding of the intrinsic material properties and their device-level implications, providing a robust foundation for exploring the optoelectronic, thermal, and photovoltaic potential of Q_3_GaBr_6_ (Q = Na and K) structure.

Overall, the computational methodology adopted in this study combines first-principles electronic-structure calculations with device-level modeling to provide a complete and reliable evaluation of Q_3_GaBr_6_ (Q = Na, K). This integrated approach ensures that both intrinsic material stability and practical photovoltaic performance are simultaneously addressed, strengthening the predictive power of the present investigation.

## 3. Findings and discussion

### 3.1. X-ray diffraction (XRD) analysis

**Fig. 1:**
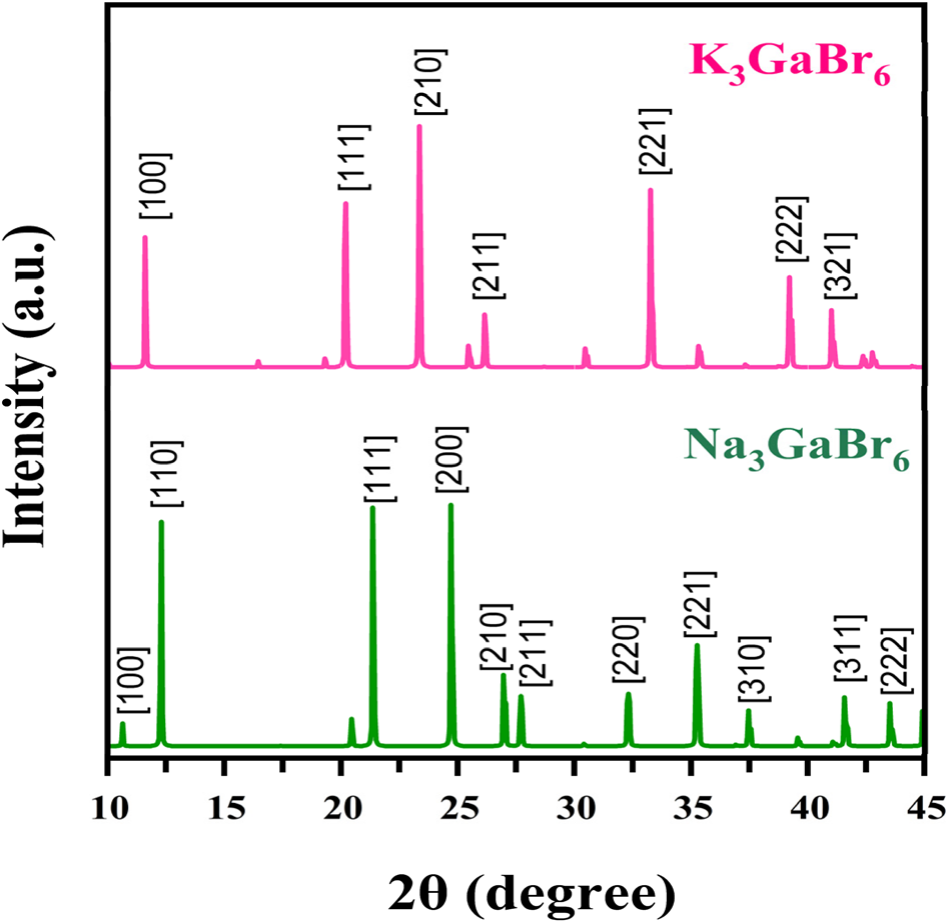
X-ray diffraction spectra of Q_3_GaBr_6_ (Q = Na and K) structure.

The simulated XRD patterns of Q_3_GaBr_6_ (Q = K and Na), obtained using CASTEP, are shown in Fig. 1 and provide essential insight into their structural characteristics. X-ray diffraction operates on the principle of constructive interference of X-rays scattered from periodic atomic planes, governed by Bragg's law *n*_λ_ = 2*d* sin *θ*, which directly relates peak positions to interplanar spacing and lattice dimensions.^38^ The diffraction profiles of both compounds exhibit sharp, well-defined peaks indicative of high crystallinity and confirm the formation of the desired cubic structure with *Fm*3̄*m* symmetry.

For Na_3_GaBr_6_ (green pattern, bottom), the prominent reflections appear at low angles around 12° to 13°, corresponding to the (100) and (110) planes, followed by intense peaks indexed to the (111), (200), (210), and (211) planes in the 20° to 27° region. Additional characteristic reflections associated with the (220), (221), (310), (311), and (222) planes are observed at higher angles, completing the diffraction fingerprint of the cubic *Fm*3̄*m* phase. For K_3_GaBr_6_ (pink pattern, top), the main diffraction peaks are indexed to the (100), (111), (210), (211), (221), (222), and (321) crystallographic planes. Compared with Na_3_GaBr_6_, all diffraction peaks of K_3_GaBr_6_ are systematically shifted toward lower 2*θ* values. This shift reflects an expansion of the unit cell, which is consistent with the substitution of the smaller Na^+^ ion (1.02 Å) by the larger K^+^ ion (1.38 Å) according to Shannon's ionic radii.^39^ The incorporation of the larger K^+^ cation increases the lattice spacing and interplanar distances, resulting in a systematic displacement of the diffraction peaks toward lower angles in K_3_GaBr_6_.

The most intense peaks for both compounds correspond to low-index planes such as (111), (200)/(210), and (221), indicating strong diffraction from densely packed atomic planes. The consistency of the peak positions with the cubic indexing and the absence of any extra reflections confirm that both materials preserve the same cubic structural symmetry while differing only in lattice size.

### 3.2. Analysis of structural properties

**Fig. 2:**
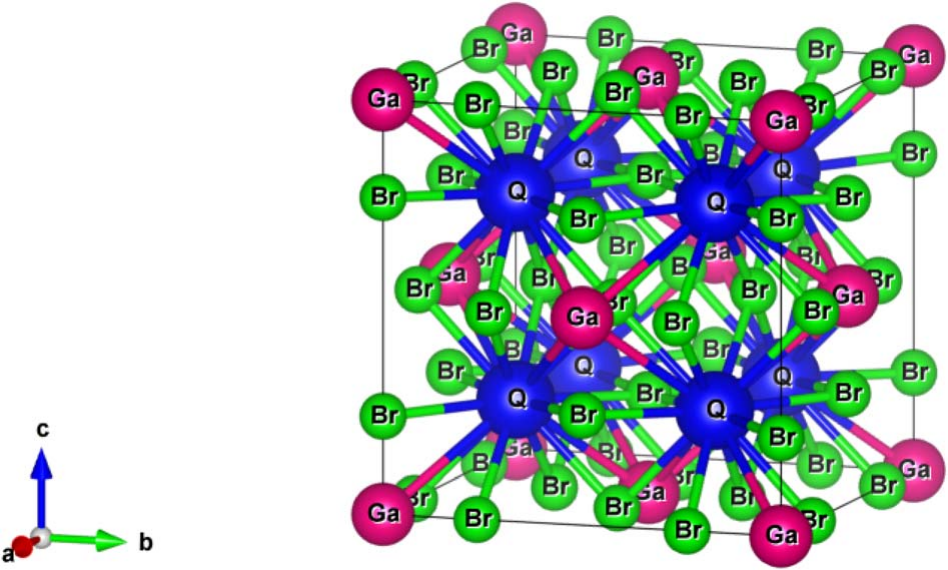
The crystal structure of Q_3_GaBr_6_ (Q = Na, K) materials.

The Q_3_GaBr_6_ (Q = Na and K) materials crystallize in the cubic *Fm*3*m* (space group 225) structure, which is characteristic of highly symmetric halide frameworks.^18^ In this configuration, the Q cations occupy the 8c Wyckoff sites at (0.25, 0.25, 0.25), while Ga resides at the 4a position (0, 0, 0), forming the center of the GaBr_6_ octahedra. The Br anions are distributed over the 24e positions (*x*, 0, 0) with a refined positional parameter of *x* = 0.236 024, ensuring the correct octahedral coordination around Ga. This atomic arrangement stabilizes the cubic lattice and highlights the structural role of the Q-site cation in dictating the symmetry of Q_3_GaBr_6_ compounds. The corresponding crystal structure is shown in Fig. 2.

The structural properties of Q_3_GaBr_6_ (Q = Na and K) show a clear dependence on both the Q-site cation size and the choice of exchange–correlation functional. As summarized in Table 1, GGA-PBE predicts a lattice constant of 7.747 Å and a unit-cell volume of 328.804 Å^3^ for Na_3_GaBr_6_, while the incorporation of the larger K^+^ ion in K_3_GaBr_6_ leads to an expanded lattice constant of 8.085 Å and a volume of 373.764 Å^3^, consistent with the expected ionic-radius-driven lattice expansion. When recalculated using the *meta*-GGA SCAN functional, both compounds exhibit noticeably reduced lattice volumes (308.729 Å^3^ for Na_3_GaBr_6_ and 355.000 Å^3^ for K_3_GaBr_6_), reflecting the well-known tendency of SCAN to yield more compact and energetically accurate structures due to its improved treatment of intermediate-range exchange–correlation effects.^40^ In terms of electronic properties the electronic band gaps of several Cs-based halide compounds (Cs_3_InI_6_, Cs_3_InCl_6_, Cs_3_InBr_6_, Cs_3_TlI_6_, Cs_3_TlCl_6_, and Cs_3_TlBr_6_) using both GGA-level and hybrid HSE06 calculations.^41^ At the GGA level, these materials exhibit moderate band gaps in the range of about 0.93 to 1.60 eV, while the corresponding HSE06 values are significantly higher, lying between ∼1.72 and 2.79 eV. This systematic increase from GGA to HSE06 highlights the well-known underestimation of band gaps by semi local functionals and the improved accuracy obtained from hybrid approaches. In our work, Na_3_GaBr_6_ and K_3_GaBr_6_ show the same band-gap trend. The GGA-PBE band gaps (1.991 and 1.445 eV) lie in the same range as those of Cs_3_QR_6_ (Q = In, Tl, Ga; R = I, Cl, Br). With more advanced functionals, the band gaps increase to 2.602 and 1.985 eV (mGGA-rSCAN) and further to 3.096 and 2.567 eV (HSE06), respectively. This systematic increase from GGA → mGGA → HSE06 is fully consistent with the behavior observed.^41^ Moreover, while mainly focuses on band-gap values,^41^ our work extends this analysis by providing a comprehensive set of structural and thermodynamic parameters, including lattice constants, unit-cell volumes, densities, and formation enthalpies. This makes our study completer and more systematic, offering not only electronic properties but also stability and structural validation, thereby strengthening the reliability and applicability of Na_3_GaBr_6_ and K_3_GaBr_6_ as promising lead-free halide materials.

**Table 1:** The energy band gap, lattice constants, unit cell volume & formation enthalpy of Q_3_GaBr_6_ (Q = Na, K) perovskites

Ref.	Compounds	GGA-PBE				mGGA-RSCAN	HSE06	Formation enthalpy, Δ*E*_f_ (eV per atom)
		Energy band gap, eV	Lattice constant *a*^0^ (Å)	Unit cell volume, *V* (Å^3^)	Density, g cm^−3^	Energy band gap, eV	Energy band gap, eV	
This Work	Na_3_GaBr_6_	1.991	7.747	328.804	3.121	2.602	3.096	−2.9357
	K_3_GaBr_6_	1.445	8.085	373.764	2.960	1.985	2.567	−2.9906
41	Cs_3_InI_6_	1.60	—	—	—	—	2.79	—
	Cs_3_InCl_6_	1.45	—	—	—	—	2.68	—
	Cs_3_InBr_6_	1.55	—	—	—	—	2.78	—
	Cs_3_TlI_6_	0.99	—	—	—	—	1.73	—
	Cs_3_TlCl_6_	0.93	—	—	—	—	1.72	—
	Cs_3_TlBr_6_	1.01	—	—	—	—	1.78	—

The formation energy (Δ*E*_f_) of a compound quantifies its thermodynamic stability with respect to decomposition into its elemental constituents. A negative formation energy indicates that the material is thermodynamically stable, whereas a positive value implies instability.^42^ For the halide perovskites Q_3_GaBr_6_ (Q = Na, K), the formation energy can be evaluated using eqn (1),

1 Δ*E*_f_ = *E*_tot_(A_3_GaBr_6_) − 3*E*(A) − *E*(Ga) − 6*E*(Br)

From a thermodynamic perspective, GGA-PBE-calculated formation enthalpies are markedly negative (−2.9357 eV per atom for Na_3_GaBr_6_ and −2.9906 eV per atom for K_3_GaBr_6_), confirming their stability and spontaneous formation tendency. The slightly more negative value for K_3_GaBr_6_ suggests marginally greater energetic favorability, potentially due to enhanced lattice relaxation enabled by the larger K^+^ cation. The combined structural compactness, moderate-to-wide band gaps, and high thermodynamic stability indicate that both Na_3_GaBr_6_ and K_3_GaBr_6_ are promising candidates for optoelectronic and related functional applications.

The Goldschmidt tolerance factor (*t*) is a classical geometric descriptor widely used to assess the formability and structural stability of perovskite and double-perovskite materials. An ideal cubic perovskite corresponds to *t* = 1, where the ionic sizes are optimally matched and lattice distortions are minimal. When 0.8 ≤ *t* ≤ 0.9, the structure is generally stable but often exhibits cooperative tilting of the GaBr_6_ octahedra, leading to orthorhombic or rhombohedral distortions. For 0.9 ≤ *t* ≤ 1.0, the perovskite typically maintains a high-symmetry cubic or slightly tetragonal phase with limited octahedral rotations. In contrast, values of *t* < 0.8 or *t* > 1.0 tend to destabilize the perovskite framework, promoting non-perovskite or hexagonal phases.^43,44^2
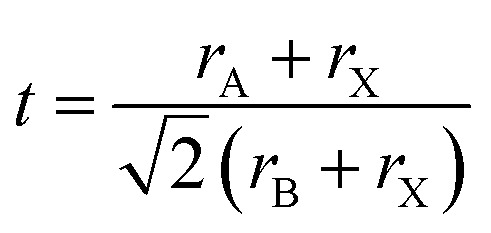


Using the ionic radii *r*_A_, *r*_B_, and *r*_X_, the calculated tolerance factor increases from 0.918 for Na_3_GaBr_6_ to 0.987 for K_3_GaBr_6_, reflecting the larger ionic radius of K^+^, as shown in Table 2. This increase shifts the structure closer to the ideal cubic limit, indicating that K_3_GaBr_6_ is likely to be more symmetric and more stable in a cubic phase compared to Na_3_GaBr_3_.

**Table 2:** Tolerance factor variation in Q_3_GaBr_6_ (Q = Na, K) perovskites

Materials	*r*_A_	*r*_B_	*r*_X_	Tolerance factor (*t*)
Na_3_GaBr_6_	1.39	0.62	1.96	0.918
K_3_GaBr_6_	1.64	0.62	1.96	0.987
Ga_2_CeCl_6_ (ref. 45)	1.3	0.97	1.81	0.791
Tl_2_CeBr_6_ (ref. 45)	1.59	0.97	1.96	0.8567

The variation of bond length within a compound refers to the differences in distances between atoms of the same bonding type, indicating that not all atoms experience identical local environments. Such variations often arise from lattice distortions, differences in ionic sizes, or symmetry breaking within the crystal structure. This is important in research because bond length variation directly influences a material's structural stability, bonding strength, and electronic properties. Shorter bonds usually indicate stronger interactions, while longer ones suggest weaker bonding or lattice strain. Understanding these variations helps researchers explain and predict changes in mechanical, optical, and electronic behavior, enabling the design of materials with optimized performance for specific applications.

Table 3 presents the bond lengths of Na_3_GaBr_6_ and K_3_GaBr_6_ compounds, revealing how cation size influences their structural geometry. In both compounds, the Ga–Br bonds are the shortest (≈2.56 to 2.57 Å), indicating strong covalent interactions within the GaBr_6_ octahedra, while the longer Na–Br and K–Br bonds suggest weaker ionic interactions. When the larger K ion replaces Na, all bond lengths increase slightly, reflecting lattice expansion due to the larger ionic radius of K^+^. The presence of multiple A–Br and A–Ga distances implies a distorted coordination environment, but the Ga–Br framework remains relatively stable. Overall, the table indicates that cation substitution from Na to K leads to an expanded and slightly distorted lattice without significantly affecting the rigid GaBr_6_ octahedral structure.

**Table 3:** The variation of bond length within the atom of Q_3_GaBr_6_ (Q = Na, K) perovskites

Compounds	Bonds	Bond length, *L* (Å)
Na_3_GaBr_6_	Na (1,2)–Br	2.911
	Na (3)–Br	3.877
	Ga–Br	2.566
	Na (1,2)–Ga	4.744
	Na (3)–Ga	5.477
K_3_GaBr_6_	K (1,2)–Br	3.144
	K (3)–Br	4.052
	Ga–Br	2.572
	K (1,2)–Ga	4.951
	K (3)–Ga	5.717

### 3.3. Electronic properties

**Fig. 3:**
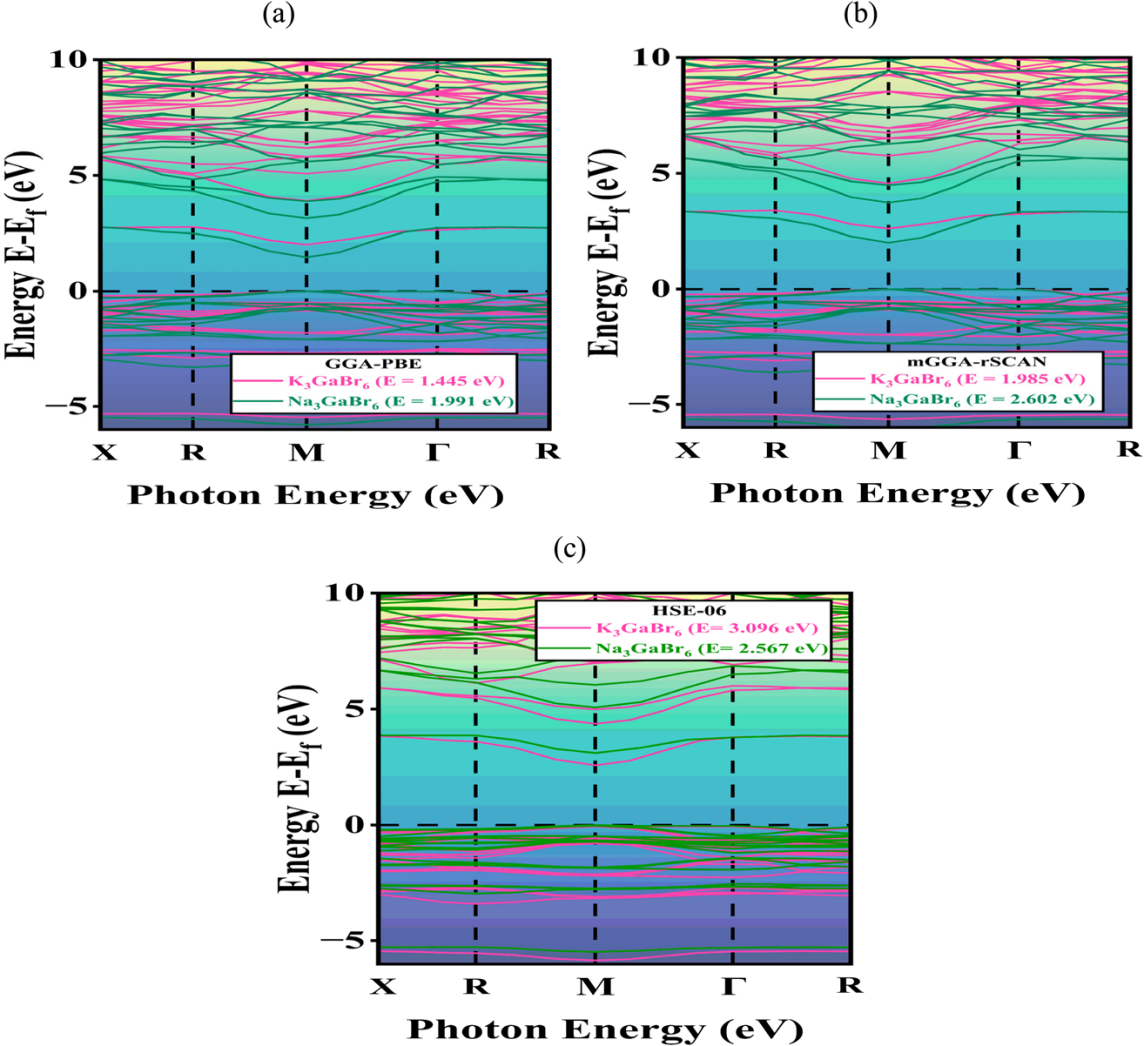
The different atomic orbitals to the valence and conduction bands of Q_3_GaBr_6_ (where A = Na, K) (a) GGA-PBE, (b) mGGA-RSCAN and (c) HSE06 with electronic band structure.

After completing the geometric optimization of Q_3_GaBr_6_ (Q = Na and K), it is essential to investigate their electronic structure to determine whether these materials exhibit semiconducting behavior and evaluate their potential suitability for photoelectric and optoelectronic applications. The electronic band structure and density of states (DOS) calculations provide fundamental insights into the energy dispersion of electrons, band-gap characteristics, and orbital contributions that govern optical absorption, carrier transport, and radiative efficiency.^46^Fig. 3 presents the band structures of Na_3_GaBr_6_ and K_3_GaBr_6_, obtained using two different exchange–correlation functionals along the high-symmetry paths. In both materials, the conduction band minimum (CBM) and valence band maximum (VBM) occur at the same symmetry point (M), confirming their direct band-gap nature. Such a direct band gap is highly advantageous, as it facilitates stronger optical absorption and efficient radiative recombination, making these compounds promising candidates for optoelectronic and photoelectric applications.^47^ Using the GGA-PBE functional (Fig. 3(a)), K_3_GaBr_6_ exhibits a conduction band minimum (CBM) of 1.445 eV and a valence band maximum (VBM) located at 0 eV, both occurring at the M high-symmetry point, yielding a direct band gap of 1.445 eV. Similarly, Na_3_GaBr_6_ shows a direct band gap of 1.991 eV at the same *k*-point. When the more advanced mGGA-rSCAN functional (Fig. 3(b)) is employed, the calculated band gaps increase to 1.985 eV for K_3_GaBr_6_ and 2.602 eV for Na_3_GaBr_6_, highlighting the well-known tendency of semi local GGA to underestimate band gaps and the improved accuracy provided by *meta*-GGA approaches. Furthermore, the hybrid HSE06 functional (Fig. 3(c)) predicts even larger band gaps of 3.096 eV for K_3_GaBr_6_ and 2.567 eV for Na_3_GaBr_6_, owing to the inclusion of a fraction of exact Hartree–Fock exchange, which significantly improves the description of electronic exchange correlation effects. The HSE06 results are generally considered closer to experimental values and therefore serve as a reliable benchmark for validating the electronic structure obtained from semi local and *meta*-GGA functionals.

The systematic increase in band-gap values from GGA-PBE to mGGA-rSCAN and further to HSE06 reflects the intrinsic limitation of semi local exchange–correlation functionals in underestimating band gaps due to the absence of derivative discontinuity in the exchange potential. The consistency of the GGA → mGGA → HSE06 trend observed in this work confirms the robustness of the calculated electronic structure and supports the reliability of the predicted semiconducting behavior of Na_3_GaBr_6_ and K_3_GaBr_6_.

Although mGGA-rSCAN and HSE06 provide more accurate band-gap estimations, both are computationally more demanding, with HSE06 being particularly expensive in terms of computational resources and time. As a result, these functionals are less practical for extensive calculations of optical, mechanical, and other physical properties on standard personal-computer platforms. In contrast, GGA-PBE offers an optimal balance between computational efficiency and acceptable quantitative accuracy. Therefore, all subsequent calculations in this work were performed using the GGA-PBE functional, while mGGA-rSCAN and HSE06 were employed mainly for band-gap correction and validation purposes.^48^

### 3.4. Density of states (DOS)

The DOS describes the number of electronic states available at each energy level in a material. It is obtained by integrating the electronic band structure over the Brillouin zone and indicates how electrons occupy different energy ranges. Peaks in the DOS correspond to energies where many states are concentrated, while low-DOS regions reflect fewer available states.^49^ The Partial Density of States (PDOS) further resolves these states into contributions from specific atoms or orbitals, helping to identify the origin of the valence and conduction bands, as well as possible hybridization between atomic orbitals. DOS and PDOS together provide essential insight into the electronic structure and bonding characteristics of the material.^50^

In both K_3_GaBr_6_ and Na_3_GaBr_6_, the valence band region (below the Fermi level, *E*_F_) is predominantly governed by the Br-4p orbitals, which exhibit strong hybridization with minor contributions from Ga-4p states.

**Fig. 4:**
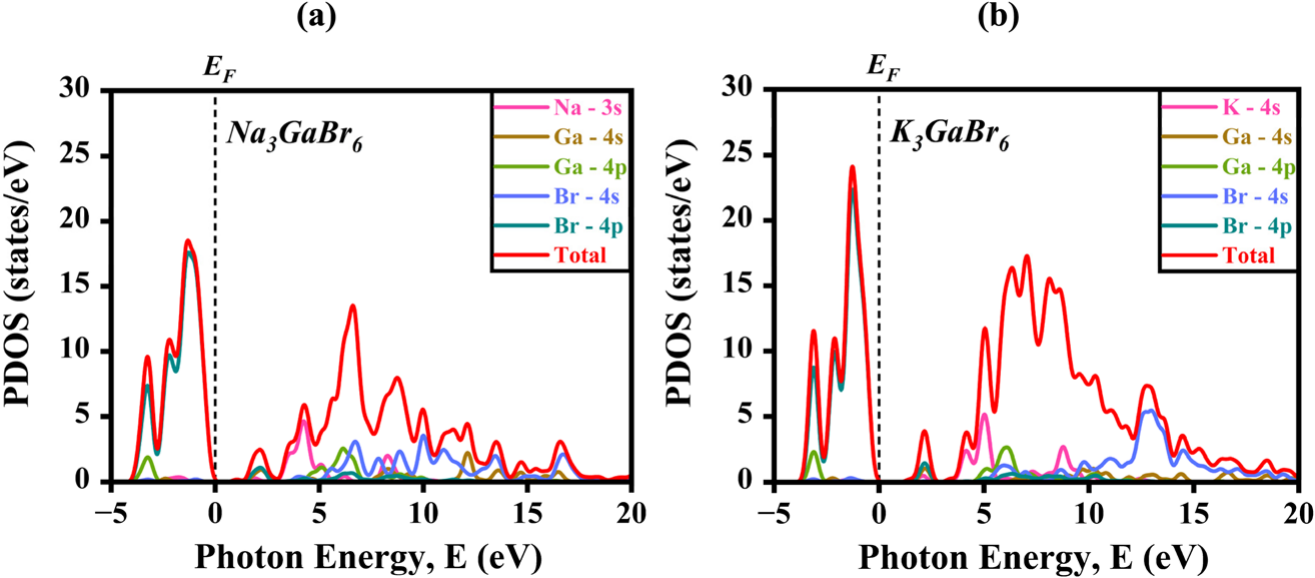
The density of states of (a) Na_3_GaBr_6_ (b) K_3_GaBr_6_ structure.

This confirms that bromine plays the dominant role in defining the bonding characteristics and shaping the valence band maximum.^51^ The conduction band region (above *E*_F_) mainly originates from the Na-3s and K-4s orbitals, along with noticeable hybridization from Br-4s states, indicating that Ga–Br interactions significantly influence the conduction band minimum and consequently determine the bandgap nature. As expected, the alkali cations (Na, K) contribute minimally to the electronic states, functioning primarily as charge-compensating species within the lattice. The corresponding PDOS profiles are illustrated in Fig. 4(a and b). For Na_3_GaBr_6_ [Fig. 4(a)], the VBM and CBM peaks appear at 18.08 eV and 13.32 eV, respectively, while for K_3_GaBr_6_ [Fig. 4(b)], the VBM and CBM peaks are observed at 23.81 eV and 17.084 eV. In both materials, the TDOS plots show valence- and conduction-band peaks extending slightly into one another, which arises from DFT-related smearing or numerical broadening parameters rather than indicating physical overlap. The calculated bandgap values obtained from the DOS are 1.991 eV for Na_3_GaBr_6_ and 1.445 eV for K_3_GaBr_6_, consistent with their electronic band structures.

### 3.5. Optical properties

Optical properties describe how a material interacts with incident light through processes such as absorption, reflection, transmission, refraction, and emission, all of which are fundamentally governed by its electronic structure. Key optical parameters include the absorption coefficient, which quantifies light absorption per unit distance; the refractive index, representing the reduction of light speed within the medium; the dielectric function, describing the material's response to external electromagnetic fields; and the optical conductivity, which characterizes the current generated under optical excitation.^52^ Understanding these properties is essential for the design and optimization of optoelectronic and photonic devices such as solar cells, photodetectors, LEDs, and optical coatings.^53^

It should be noted that the present optical property calculations are performed within the independent-particle approximation using standard DFT, where excitonic effects arising from electron–hole interactions are not explicitly included. In halide perovskites, excitonic contributions can be significant, particularly near the absorption edge, and may lead to a red shift and enhancement of optical absorption. Therefore, the calculated optical spectra represent the intrinsic interband transition behavior of the materials and may slightly underestimate excitonic features. A more accurate description would require many-body approaches such as the GW approximation combined with the Bethe–Salpeter equation (GW-BSE), which are computationally demanding and beyond the scope of the present work.

#### 3.5.1. Dielectric function

**Fig. 5:**
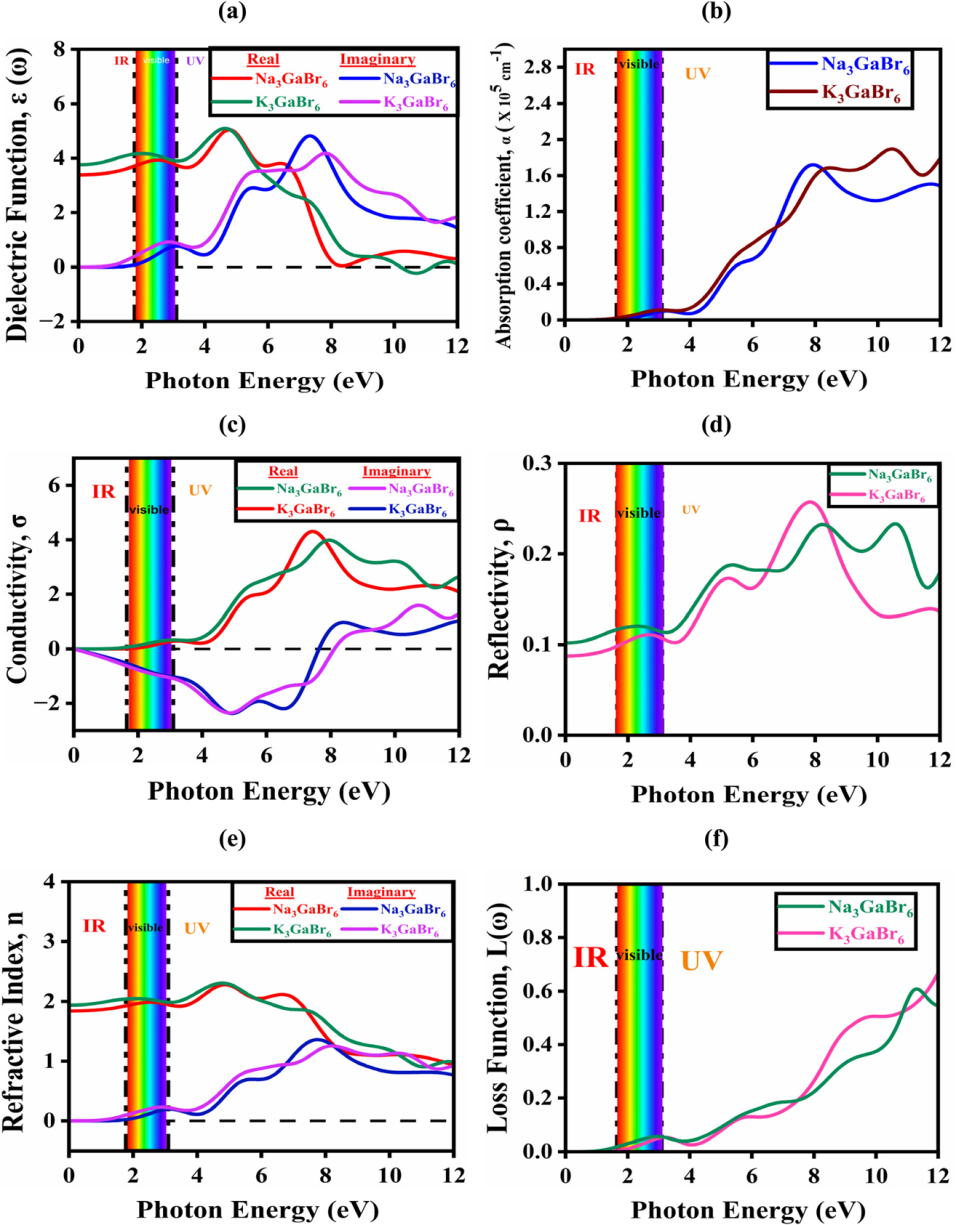
Calculated optical functions of Q_3_GaBr_6_ (A = Na, K) perovskites: (a) dielectric function, (b) absorption coefficient, (c) optical conductivity, (d) reflectivity, (e) refractive index, and (f) energy loss function.

The dielectric spectra of Q_3_GaBr_6_, presented in Fig. 5(a), describe their interaction with incident electromagnetic radiation. The dielectric function is a complex quantity and is divided into two components: the real part (*ε*_1_) and the imaginary part (*ε*_2_). The real part *ε*_1_(*ω*) describes the polarization response of the material to the incident electromagnetic field and is associated with the ability of the system to store electric energy. It is directly related to the refractive index and provides information about the dispersion behavior of the material. On the other hand, the imaginary part *ε*_2_(*ω*) represents the absorption of electromagnetic radiation and originates from interband electronic transitions. It is directly connected to the energy dissipation inside the material and determines its optical absorption and photoconductivity.^45,54,55^ The dielectric function, defined as,

3 *ε*(*ω*) = *ε*_1_(*ω*) + *jε*_2_(*ω*)

The real part, *ε*_1_(*ω*) is obtained using the Kramers–Kronig relation:4
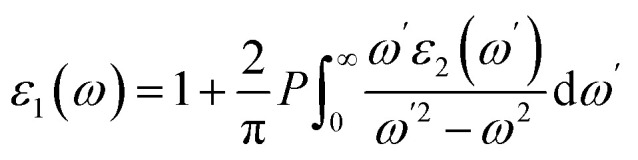


At zero photon energy, the real part *ε*_1_(*ω*) of Na_3_GaBr_6_ begins at approximately 3.39 and increases to a maximum value of about 5.02 near 4.8 eV, indicating strong polarization and enhanced photon-matter interaction. Beyond 8.32 eV, *ε*_1_(*ω*) decreases and becomes zero to negative, suggesting the emergence of plasmonic-like or high-reflectivity behavior at higher photon energies. In comparison, K_3_GaBr_6_ exhibits a slightly higher static dielectric constant with an initial *ε*_1_(0) value of approximately 3.75, and reaches its maximum of about 5.09 around 4.72 eV. With further increase in photon energy, *ε*_1_(*ω*) gradually decreases and crosses zero near 10.27 eV, becoming negative thereafter. Overall, while both compounds demonstrate similar dielectric characteristics, Na_3_GaBr_6_ shows the onset of negative dielectric behavior at a lower energy than K_3_GaBr_6_, indicating comparatively stronger plasmonic tendencies in the high-energy region. The spectrum also highlights the behavior of the imaginary part *ε*_2_(*ω*), which is critical for understanding the optical absorption properties. The imaginary component is calculated from the momentum-matrix elements between occupied and unoccupied states as,5
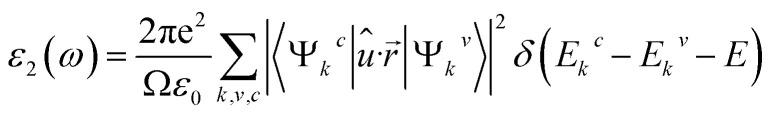


The imaginary part of the dielectric function, *ε*_2_(*ω*) starts to rise at photon energies of about 1.59 eV for Na_3_GaBr_6_ and 1.06 eV for K_3_GaBr_6_, corresponding to the onset of optical absorption in these materials. Within the visible energy range (1.65 to 3.1 eV), both compounds exhibit distinct absorption peaks, with Na_3_GaBr_6_ showing a peak value of approximately 0.92 at 2.72 eV and K_3_GaBr_6_ exhibiting a peak of about 0.76 at 3.10 eV. As the photon energy further increases into the ultraviolet region, the intensity of *ε*_2_(*ω*) increases significantly, reaching maximum values of about 4.82 at 7.30 eV for Na_3_GaBr_6_ and 4.21 at 7.80 eV for K_3_GaBr_6_. Although Na_3_GaBr_3_ shows stronger optical transitions, the lower absorption onset and extended response toward lower photon energies in K_3_GaBr_6_ suggest comparatively better suitability for optoelectronic applications, particularly in devices requiring enhanced low-energy light absorption.

#### 3.5.2. Absorption coefficient (*α*)

The absorption coefficient quantifies the attenuation of incident radiation per unit path length as a function of photon energy.^56^ Within the DFT framework, it is evaluated from the complex dielectric function according to6
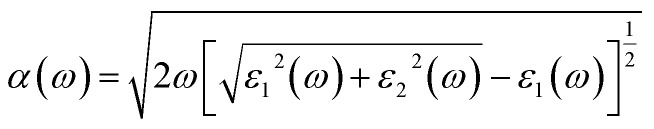


Fig. 5(b) presents the absorption spectra of Na_3_GaBr_6_ and K_3_GaBr_6_, illustrating their spectral response and light-harvesting capability over a wide photon-energy range. This optical parameter is crucial for the design of optoelectronic devices such as solar cells, ultraviolet (UV) photodetectors, and photodiodes, where strong and efficient photon absorption is required. In the infrared region (0–1.5 eV), both compounds show negligible absorption, indicating minimal photon interaction due to the absence of available electronic transitions below the band gap. In the visible energy range (1.65 to 3.10), the absorption coefficients exceed 10^4^ cm^−1^ for both compounds, signifying the onset of strong interband electronic transitions from the valence to the conduction band, which is essential for efficient solar light harvesting.^57,58^ Specifically, absorption peaks of approximately 0.98 × 10^4^ cm^−1^ for Na_3_GaBr_6_ and 1.12 × 10^4^ cm^−1^ for K_3_GaBr_6_ are observed near 3.10 eV, indicating effective interaction with visible photons and strong electronic transitions. In the ultraviolet region, the absorption intensifies significantly, reaching maximum values of about 1.71 × 10^5^ cm^−1^ at 7.89 eV for Na_3_GaBr_6_ and 1.88 × 10^5^ cm^−1^ at 10.57 eV for K_3_GaBr_6_. This strong UV absorption originates from high-energy electronic transitions involving deeper valence states, highlighting their suitability for UV-sensitive optoelectronic applications. Overall, the absorption behavior directly reflects the electronic band structure of these materials, with K_3_GaBr_6_ showing comparatively stronger high-energy absorption.

#### 3.5.3. Conductivity (*σ*)

The optical conductivity *σ* represents a material's ability to conduct current when a material is exposed to photon-induced current.^59,60^ Both the real and imaginary part of this property offers insights into the charge carrier dynamics and interband transitions of a material. The calculated value of the reflectivity of K_3_GaBr_6_ and Na_3_GaBr_6_ is depicted in Fig. 5(c), revealing their potential for optoelectronic and photovoltaic applications by indicating how they interact with light across the infrared, visible, and ultraviolet spectra. The calculated real part of the conductivity spectra of K_3_GaBr_6_ and Na_3_GaBr_6_ are presented in Fig. 5(c), illustrating their interaction with electromagnetic radiation across the infrared (IR), visible, and ultraviolet (UV) regions and underscoring their relevance for optoelectronic and photovoltaic applications. The reflectivity of both materials begins to rise near 1.33 eV, indicating the onset of electronic transitions. In the visible region, pronounced peaks are observed at approximately 0.32 for Na_3_GaBr_6_ and 0.31 for K_3_GaBr_6_ around 3.10 eV, reflecting moderate optical losses and efficient photon coupling. As the photon energy increases into the UV region, the *σ*_1_ intensifies markedly, reaching maximum values of about 3.97 at 8.04 eV for Na_3_GaBr_6_ and 4.30 at 7.40 eV for K_3_GaBr_6_, with K_3_GaBr_6_ exhibiting comparatively stronger UV reflectivity. In addition, the imaginary part of the optical conductivity, *σ*_2_(*ω*), for both compounds exhibits negative values in the IR and visible ranges, signifying a predominantly reactive optical response with minimal energy dissipation. The *σ*_2_(*ω*) curves approach zero near 8.08 eV for Na_3_GaBr_6_ and 7.62 eV for K_3_GaBr_6_ in the UV region, indicating the transition toward strong interband absorption. This behavior is commonly associated with dielectric-to-conducting characteristics or pronounced excitonic effects at higher excitation energies. Furthermore, both materials display deep negative minima in the visible region (approximately −2.35), which implies reduced absorption losses and improved optical transparency, benefiting applications that require low-loss light transmission.

#### 3.5.4. Reflectivity (*ρ*)

Reflectivity (*ρ*) is an important optical characteristic that assesses the fraction of incoming light that is bounced back from a material's surface, represented as a ratio between 0 (no reflection) and 1 (complete reflection).^61^ Reflectivity, shown in Fig. 5(d), characterizes how a material interacts with incident radiation across different photon-energy regions and is therefore a key parameter for assessing its suitability for optoelectronic and photonic applications. It is directly derived from the complex dielectric function using the relation:7
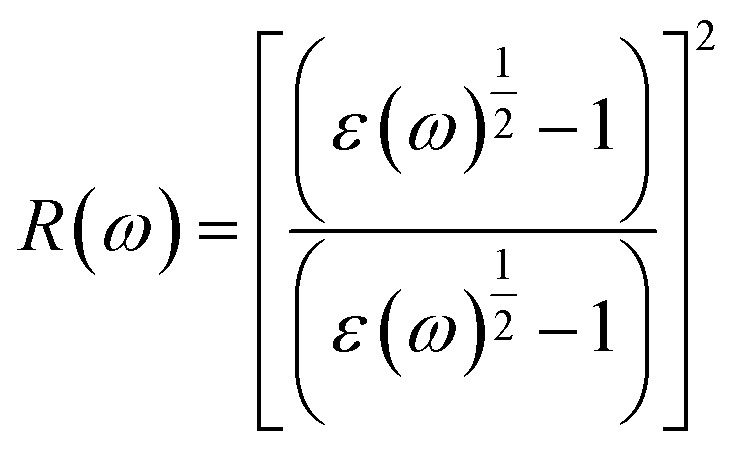


In the infrared (IR) region (0–1.65 eV), both compounds exhibit low reflectivity values of about 0.116 for Na_3_GaBr_6_ and 0.097 for K_3_GaBr_6_ at 1.65 eV, as shown in Fig. 5(d), indicating minimal surface reflection and good optical transparency. As the photon energy enters the visible range (1.65–3.10 eV), a slight increase in reflectivity is observed in Fig. 5(d), with peak values of approximately 0.121 at 2.35 eV for Na_3_GaBr_6_ and 0.111 at 2.65 eV for K_3_GaBr_6_, where Na_3_GaBr_6_ exhibits marginally higher reflectance. In the ultraviolet (UV) region (>3.10 eV), a pronounced rise in reflectivity is also evident in Fig. 5(d), reflecting stronger photon–electron interactions at higher energies. Since the objective of this study is to identify efficient solar-cell absorber materials, the consistently low reflectivity across the visible and near-IR regions shown in Fig. 5(d) indicates enhanced light coupling and reduced optical losses. Consequently, both compounds are promising candidates as absorber layers for photovoltaic devices.

#### 3.5.5. Refractive index (*n*)

How much light slows down and bends when it enters a material is quantified by the refractive index.^62^ This optical parameter consists of a real part, *n*(*ω*), which represents the phase velocity of light in the medium, and an imaginary part, *k*(*ω*), which describes light absorption or attenuation. The refractive index can be derived from the complex dielectric function using the relation,^63^8
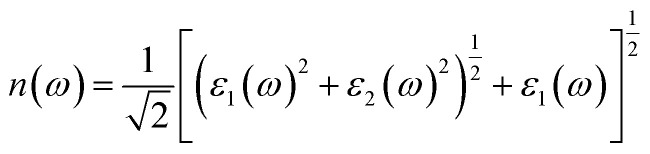
9
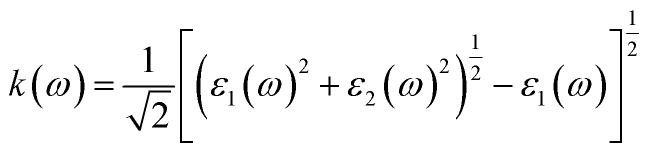


As shown in Fig. 5(e), the static refractive index *n*(0) is 1.84 for Na_3_GaBr_6_ and 1.98 for K_3_GaBr_6_, indicating moderate optical polarization, with the higher value for K_3_GaBr_6_ suggesting stronger electronic polarizability due to its larger cation size. Visible-range peaks appear at 2.44 eV (*n*(*ω*) = 1.98) for Na_3_GaBr_6_ and 1.77 eV (*n*(*ω*) = 2.04) for K_3_GaBr_6_, attributed to band-edge electronic transitions. In the UV region (3.2 to 12 eV), *n*(*ω*) increases further, reaching maxima of 2.28 and 2.30, respectively, due to strong interband excitations. The extinction coefficient *k*(*ω*) remains nearly zero in the IR-visible range, confirming low optical loss and high transparency. Absorption begins at about 1.99 eV for Na_3_GaBr_6_ and 1.45 eV for K_3_GaBr_6_, consistent with band-gap energies. Strong UV peaks (1.35 at 7.68 eV and 1.25 at 8.34 eV) arise from deeper interband transitions, indicating potential for UV-optoelectronic applications.^64^

#### 3.5.6. Loss function

Optical loss function describes energy loss due to absorption and collective electronic excitations such as plasmons and interband transitions, serving as a key indicator of high-energy electronic response.^65^ Loss function defined as,10
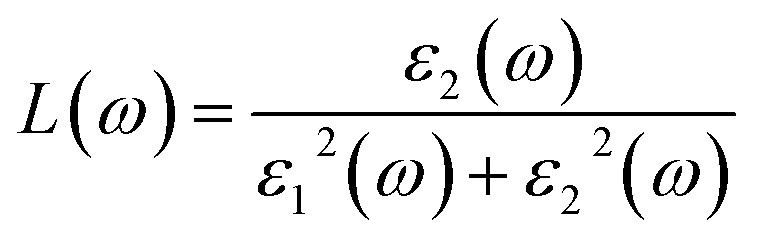


Fig. 5(f) shows the loss function of Na_3_GaBr_6_ and K_3_GaBr_6_ over the photon-energy range of 0–12 eV. In the infrared region (0 to 1.65 eV), both materials exhibit negligible optical loss, confirming high transparency and minimal energy dissipation. In the visible region, small maxima appear at 2.50 eV (0.54) for Na_3_GaBr_6_ and at 3.21 eV (0.525) for K_3_GaBr_6_, though the overall loss remains low, indicating good optical performance in this spectrum.^66^ As the photon energy enters the ultraviolet region, the loss function increases significantly, indicating the onset of strong electronic excitations. K_3_GaBr_6_ reaches a maximum value of 0.72 at 11.9 eV, while Na_3_GaBr_6_ exhibits a slightly lower peak of 0.63 near 11.3 eV. These peaks correspond to the plasma resonance frequency, where collective oscillations of conduction electrons strongly interact with incident electromagnetic radiation.^59^ The earlier and stronger plasmonic response of K_3_GaBr_6_ suggests higher electronic polarizability compared with Na_3_GaBr_6_.

### 3.6. Elastic and mechanical properties

#### 3.6.1. Elastic properties

Elastic constants describe a material's response to applied stress and are essential for evaluating mechanical stability and rigidity. For cubic perovskites, it is sufficient to determine the three independent elastic constants *C*_11_, *C*_12_, and *C*_44_ (in GPa) for both compounds considered in this study. These constants are obtained by relating the variation of the total energy to small, symmetry-adapted lattice deformations within the framework of linear elastic theory. The mechanical stability of cubic crystals is further verified using the Born stability criteria, which require:^67^

11 *C*_11_ > 0,4*C*_44_ > 0, *C*_11_ − *C*_12_ > 0 and *C*_11_ + 2*C*_12_ > 0

**Fig. 6:**
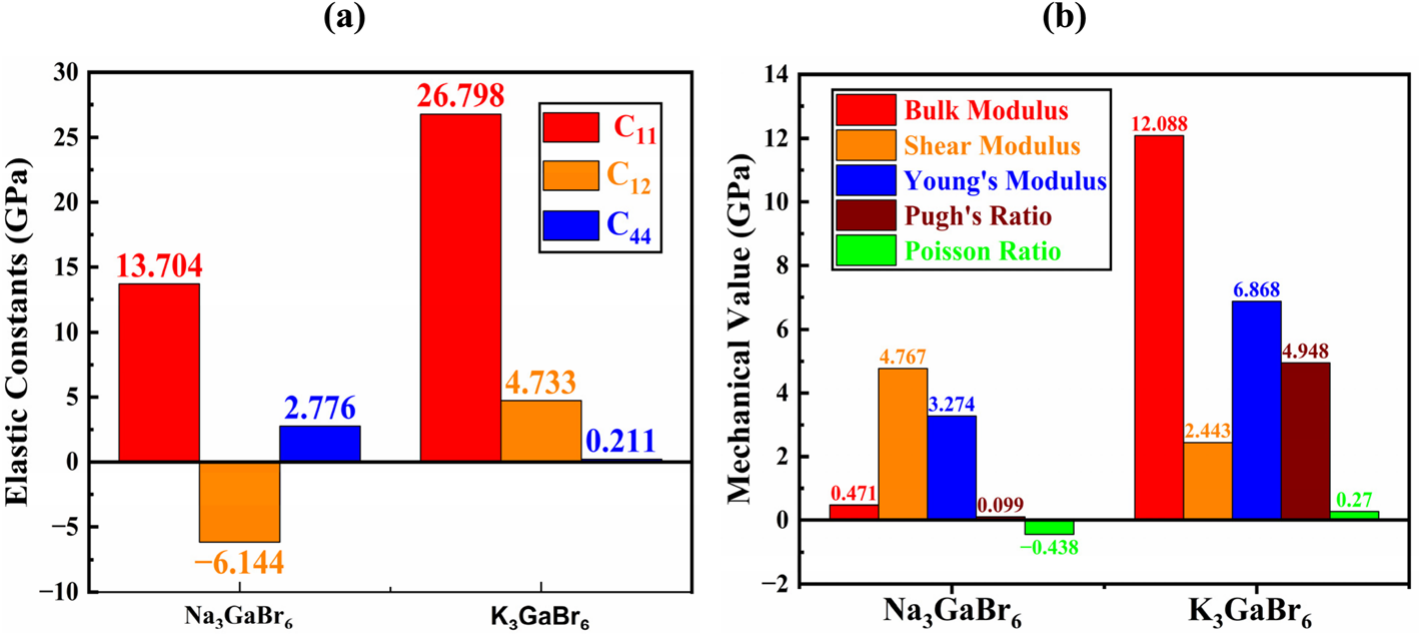
(a) Elastic constants and (b) mechanical properties of Q_3_GaBr_6_ materials.

The mechanical stability of Na_3_GaBr_6_ and K_3_GaBr_6_ was evaluated using the Born stability criteria for cubic systems. For Na_3_GaBr_6_ and K_3_GaBr_6_, the calculated elastic constants satisfy all stability conditions: *C*_11_ = 13.704 and 26.798 GPa, *C*_11_ − *C*_12_ = 19.848 and 22.065 GPa, *C*_44_ = 2.776 and 0.211 GPa, and *C*_11_ + 2*C*_12_ = 1.424 and 36.264 GPa, respectively, as shown in Fig. 6(a). The fulfillment of these inequalities (*C*_11_ > 0, 4*C*_44_ > 0, *C*_11_ − *C*_12_ > 0 and *C*_11_ + 2*C*_12_ > 0) confirms that both compounds are mechanically stable. Comparatively, K_3_GaBr_6_ exhibits a higher *C*_11_ value, indicating stronger resistance to uniaxial compression, whereas the lower *C*_44_ value of K_3_GaBr_6_ suggests reduced resistance to shear deformation compared with Na_3_GaBr_6_. These results indicate that Na_3_GaBr_6_ is relatively more resistant to shear stress, while K_3_GaBr_6_ demonstrates greater compressive stiffness. Physically, the higher *C*_11_ value of K_3_GaBr_6_ indicates stronger resistance to uniaxial compression along the principal crystallographic directions. The negative *C*_12_ value for Na_3_GaBr_6_ suggests an unusual lateral strain response under axial loading, which is often associated with auxetic-like behavior and requires careful interpretation.

In contrast, the positive *C*_12_ of K_3_GaBr_6_ reflects a more conventional elastic response. Furthermore, the much lower *C*_44_ value of K_3_GaBr_6_ indicates weaker resistance to shear deformation, implying a comparatively softer lattice under shear stress. Overall, Na_3_GaBr_6_ appears more resistant to shear distortion, while K_3_GaBr_6_ exhibits greater compressive stiffness.

#### 3.6.2. Mechanical properties

The mechanical behavior of materials is evaluated using elastic constants and derived moduli, which describe resistance to deformation under applied stress.^50,68^ The bulk modulus (*B*) represents resistance to volume change under pressure, the shear modulus (*G*) reflects resistance to shape distortion, and Young's modulus (*Y*) indicates stiffness against tensile or compressive strain 69. These moduli are obtained using the Voigt–Reuss–Hill (VRH) approximation to ensure reliable average mechanical parameters. The mechanical parameters are expressed through the following equations.12
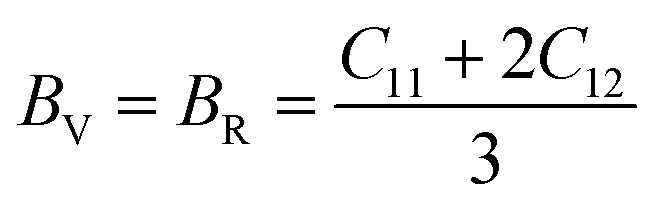
13
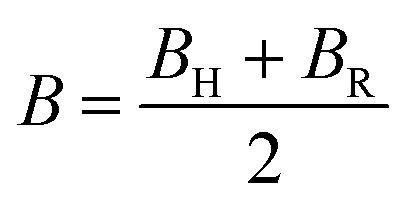
14
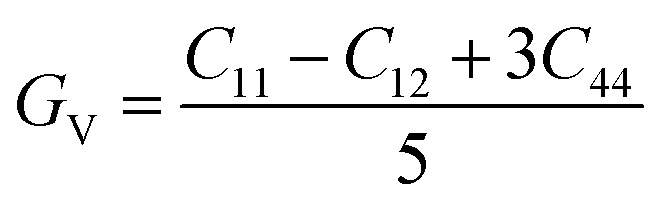
15
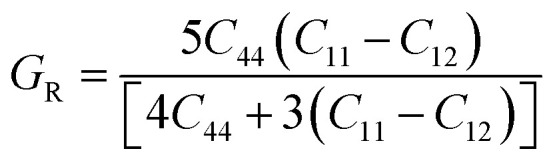
16
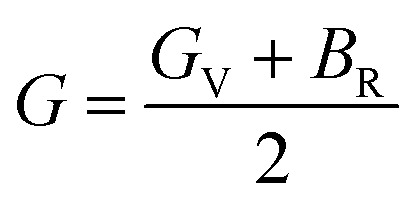
17
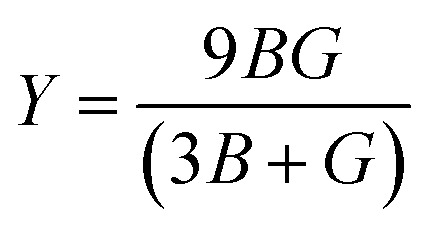


As shown in Fig. 6(b), the extremely low bulk modulus of Na_3_GaBr_6_ (0.471 GPa) reflects its highly compressible lattice and originates from the weak ionic interaction between Na^+^ and Br^−^ ions, combined with the open framework formed by isolated GaBr_6_ octahedra. Such a structure can undergo significant volume reduction under small external pressure, which naturally results in a low resistance to hydrostatic compression. In contrast, K_3_GaBr_6_ exhibits stronger interatomic cohesion and a more compact framework, leading to a much higher bulk modulus (12.088 GPa).

However, despite its high compressibility, Na_3_GaBr_6_ shows a comparatively larger shear modulus (4.767 GPa) than K_3_GaBr_6_ (2.443 GPa), indicating better resistance to shape distortion and shear deformation. This contrasting behavior suggests that while Na_3_GaBr_6_ is easily compressible under hydrostatic pressure, its lattice maintains appreciable rigidity against shear stresses due to the directional strength of the Ga–Br bonds within the rigid GaBr_6_ octahedra. Conversely, the more compact structure of K_3_GaBr_6_ enhances its resistance to volume compression but reduces its ability to withstand shear deformation. This anisotropic mechanical response highlights the distinct deformation mechanisms operating in the two compounds and underscores the strong influence of lattice topology and bonding nature on their elastic behavior. Likewise, Young's modulus is higher for K_3_GaBr_6_ (6.868 GPa) compared with Na_3_GaBr_6_ (3.274 GPa), confirming greater stiffness and tensile strength.

The Poisson's ratio (*ν*) describes the transverse strain response under axial loading and provides insight into the nature of chemical bonding. Typically, values close to 0.25 indicate mixed ionic-covalent bonding. The strongly negative Poisson's ratio in Fig. 6(b) obtained for Na_3_GaBr_6_ (*ν* = −0.438) indicates auxetic behavior, in which the material expands laterally under tensile strain. Such behavior, although uncommon, has been reported in framework-type materials and structures composed of rigid polyhedral units connected through flexible linkages. In Na_3_GaBr_6_, the rigid GaBr_6_ octahedra are interconnected by comparatively weak Na–Br ionic bonds, forming an open structural network that can accommodate transverse expansion through rotation and hinging of the octahedra under applied stress. This structural flexibility provides a plausible microscopic origin for the predicted auxetic response.18
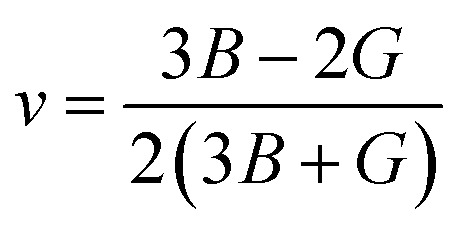


Pugh's ratio (*B*/*G*) is widely used to assess ductility, with values greater than 1.75 indicating ductile behavior and lower values signifying brittleness. The calculated *B*/*G* ratios are 0.099 for Na_3_GaBr_6_ and 4.948 for K_3_GaBr_6_, indicating that Na_3_GaBr_6_ is strongly brittle, whereas K_3_GaBr_6_ shows pronounced ductile character. These results suggest that K_3_GaBr_6_ is mechanically more suitable for flexible device fabrication, while Na_3_GaBr_6_ may be more prone to mechanical failure under stress.

Mechanical parameters derived from elastic constants provide valuable insight into a material's rigidity, deformation resistance, and structural reliability in device environments. Hardness reflects resistance to permanent deformation, while the machinability index indicates ease of mechanical processing.19
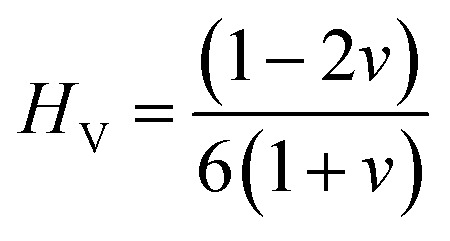
20
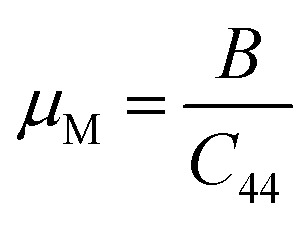


Table 4 presents the calculated mechanical parameters of Na_3_GaBr_6_ and K_3_GaBr_6_ materials, Na_3_GaBr_6_ exhibits significantly higher hardness (52.495) than K_3_GaBr_6_ (13.413), indicating stronger resistance to surface deformation and wear. The machinability index is much lower for Na_3_GaBr_6_ (0.17) than for K_3_GaBr_6_ (57.289), suggesting that K_3_GaBr_6_ is easier to machine and mechanically process.

**Table 4:** Various mechanical properties of Q_3_GaBr_6_ (Q = Na and K) materials

Mechanical properties	Na_3_GaBr_6_	K_3_GaBr_6_
Hardness (*H*)	52.495	13.413
Machinability index (*µ*_M_)	0.17	57.289
Elastic Debye temperature	52.772	112.484
Average sound velocity (*V*_m_ (m s^−1^))	593.528	1212.156
Anisotropy (*A*^U^)	2.225	60.279
Zener isotropic factor (*A*)	0.28	0.0019
Equivalent Zener anisotropy measure (*A*^eq^)	3.574	52.213
Anisotropy in share (*A*^G^)	0.182	0.858

The elastic Debye temperature is associated with lattice vibration behavior and bond strength, with higher values implying stronger interatomic interactions. The average sound velocity correlates with elastic stiffness and phonon transport. The larger Debye temperature (112.484 K) and sound velocity (1212.156 m s^−1^) of K_3_GaBr_6_ imply stronger bonding and lattice stiffness compared with Na_3_GaBr_6_ (52.772 K and 593.528 m s^−1^).

To assess the directional dependency of mechanical properties in Q_3_GaBr_6_ (Q = Na and K) perovskites, various anisotropy factors were calculated, including the universal anisotropy index (*A*^u^), Zener anisotropy factor (*A*), equivalent Zener anisotropy (*A*^eq^), and shear anisotropy index (*A*^G^). These parameters reveal the extent to which a material's elastic response varies with direction, and help identify potential weaknesses in structural applications shown in Table 4.21
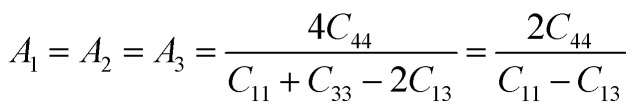


The Zener isotropic factor *A* can be defined as,22
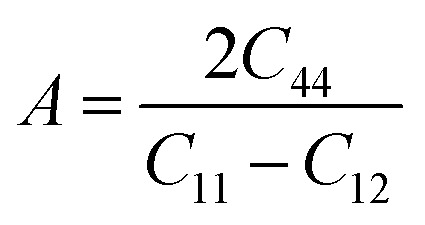


For an isotropic material, *A* = *A*_1_ = *A*_2_ = *A*_3_ = 1, and the variation from unity corresponds to the anisotropy of a material.^70^23
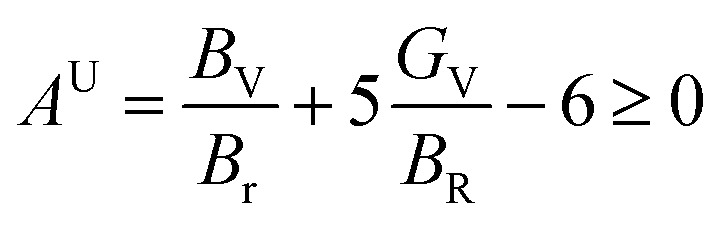
24
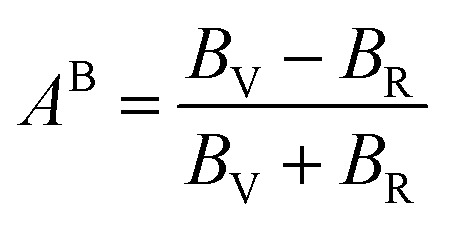
25
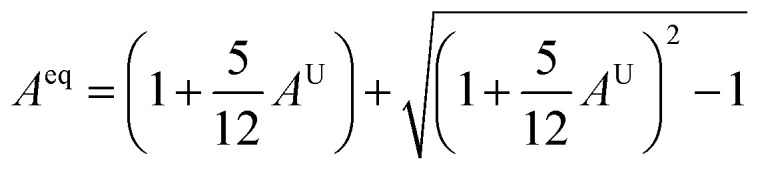
26
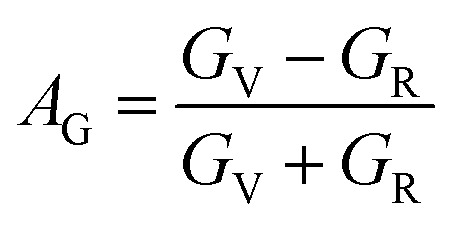


Anisotropy analysis further shows that K_3_GaBr_6_ is highly anisotropic, with a universal anisotropy index *A*^U^ = 60.279, Zener factor *A* = 0.0019, and equivalent anisotropy measure *A*^eq^ = 52.213, while Na_3_GaBr_6_ displays comparatively lower anisotropy (*A*^U^ = 2.225, *A* = 0.28, *A*^eq^ = 3.574). This indicates that Na_3_GaBr_6_ possesses a more uniform mechanical response along different crystallographic directions, whereas K_3_GaBr_6_ exhibits strong directional dependence. The lower shear anisotropy index *A*_G_ of Na_3_GaBr_6_ (0.182) compared to K_3_GaBr_6_ (0.858) further confirms its superior mechanical isotropy. Consequently, Na_3_GaBr_6_ shows higher hardness, stiffness, and directional stability, while K_3_GaBr_6_ is comparatively softer, more ductile, and easier to machine but strongly anisotropic.

**Fig. 7:**
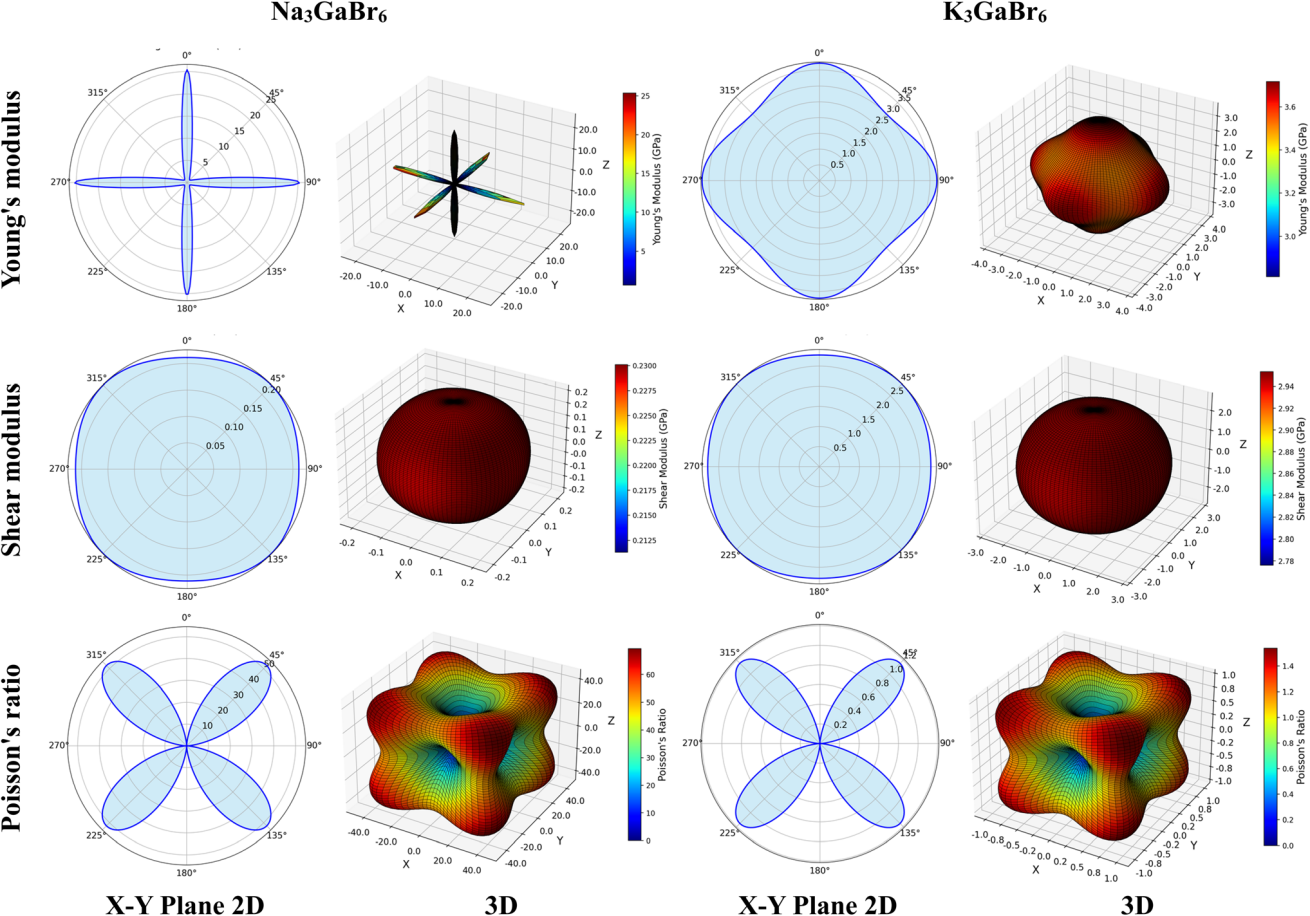
Graphical assessment of directional anisotropy showing 2D and 3D figures of Q_3_GaBr_6_ materials.

The auxetic behavior predicted for Na_3_GaBr_6_ is consistent with its elastic characteristics and structural flexibility. The finite universal anisotropy index (*A*^U^ = 2.225) together with the directional variation of Poisson's ratio shown in Fig. 7 reflects the presence of deformation pathways that allow transverse expansion under applied strain. Importantly, the absence of imaginary phonon modes confirms that this response arises from an intrinsically stable lattice, where the rigid GaBr_6_ octahedra can undergo rotational and hinging motions mediated by weak Na–Br interactions, rather than from any mechanical or dynamical instability.

### 3.7. Correlation between structural and elastic properties

The elastic behavior of Q_3_GaBr_6_ (Q = Na, K) is strongly governed by their underlying structural characteristics. The increase in lattice constant and unit-cell volume from Na_3_GaBr_6_ to K_3_GaBr_6_, caused by the larger ionic radius of K^+^, leads to a more expanded crystal framework with longer A–Br and A–Ga bond lengths. This structural expansion reduces resistance to shear deformation, which is reflected in the significantly lower *C*_44_ and shear modulus of K_3_GaBr_6_ compared to Na_3_GaBr_6_, while simultaneously enhancing its resistance to volume compression as indicated by the higher bulk modulus. Furthermore, the tolerance factor increases from 0.918 for Na_3_GaBr_6_ to 0.987 for K_3_GaBr_6_, shifting the structure closer to an ideal cubic geometry. This improved geometrical stability promotes ductile behavior in K_3_GaBr_6_, as confirmed by its large *B*/*G* ratio and positive Poisson's ratio. In contrast, the relatively smaller lattice constant and shorter bond lengths in Na_3_GaBr_6_ result in stronger directional bonding, higher shear modulus, and greater hardness, but also lead to brittle behavior and negative Poisson's ratio. Therefore, the observed elastic and mechanical responses can be directly correlated with the structural parameters: lattice expansion favors ductility and compressive stiffness, whereas structural compactness enhances shear rigidity and hardness. This strong structure property relationship highlights how Q-site cation substitution serves as an effective strategy to tune the mechanical performance of vacancy-ordered halide perovskites for device applications.

### 3.8. Anisotropy

To better understand the anisotropic mechanical behavior of Q_3_GaBr_6_ (Q = Na and K), both 2D and 3D visualizations of directional elastic properties, Young's modulus, shear modulus, and Poisson's ratio, were analyzed.

In Fig. 7 reveal how, each property varies with crystallographic direction, offering insights into the degree of anisotropy. Na_3_GaBr_6_ shows highly anisotropic behavior, particularly in Young's modulus and Poisson's ratio, with sharp directional variations and pronounced surface lobes. In contrast, K_3_GaBr_6_ displays more moderate anisotropy with relatively smoother 3D profiles. The influence of the Q-site cation is evident in shaping the directional stiffness and flexibility of the compounds. Additionally, the symmetry and shape of the 3D surfaces align with the underlying crystal structure, while 2D projections aid in identifying principal directions and extrema. These anisotropic traits are crucial for evaluating the suitability of these materials in directional stress-sensitive device applications.

### 3.9. Phonon analysis

Phonon stability analysis is employed to evaluate the dynamical stability of crystalline materials by investigating their lattice vibrations, where atoms oscillate around their equilibrium positions and their collective motions are quantized as phonons. These phonons play a fundamental role in governing the thermal, mechanical, and structural properties of solids.^71^

**Fig. 8:**
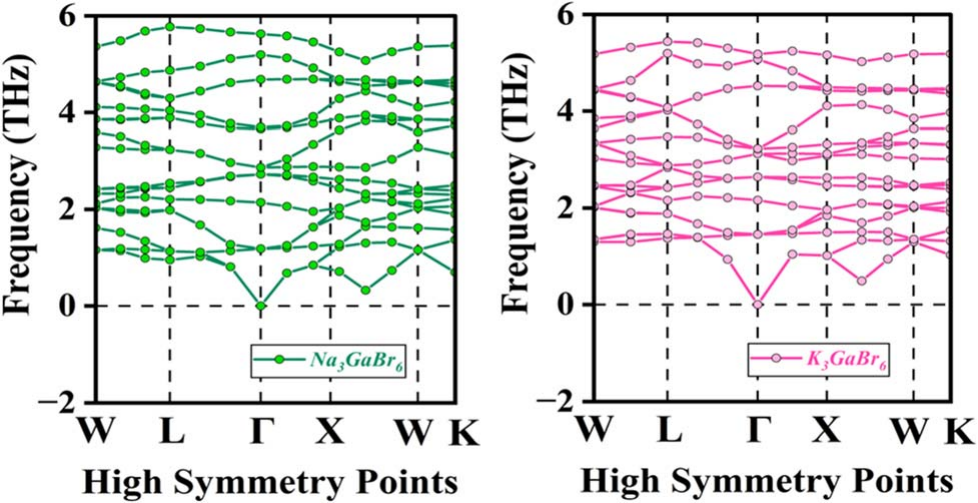
Phonon dispersion analysis of Q_3_GaBr_6_ (Q = Na and K) materials.

In lattice dynamics, phonon frequencies and vibrational modes are obtained by solving the eigenvalue problem of the dynamical matrix, which is constructed from the second-order derivatives of the total energy with respect to atomic displacements. A material is considered dynamically stable only when all phonon frequencies are real and positive throughout the entire Brillouin zone.^72^ The presence of imaginary (negative) frequencies signifies lattice instability and indicates the tendency of the system toward structural distortion or a possible phase transition.^73^ Therefore, the phonon spectrum provides direct evidence of the dynamical stability of a material, where the absence of imaginary modes confirms its structural robustness. Moreover, phonons are responsible for key physical properties such as thermal conductivity, heat capacity, electron phonon coupling, and lattice-mediated phase transitions. The phonon band structures reveal that Q_3_GaBr_6_ (Q = Na and K) materials are dynamically stable, as no imaginary (negative) phonon frequencies are detected along any high-symmetry directions (*W*–*L*–*Γ*–*X*–*W*–*K*) in the Brillouin zone, as shown in Fig. 8. This confirms that the optimized crystal structures correspond to local minima on the potential energy surface. The acoustic phonon branches originate from zero frequency at the *Γ* point, in agreement with the translational invariance of the lattice and the satisfaction of basic mechanical stability conditions. The optical phonon modes extend up to approximately 6 THz, reflecting moderate interatomic force constants in the studied systems. Notably, a large number of phonon branches are concentrated in the frequency range between 1 and 5.5 THz, indicating strong vibrational interactions among the constituent atoms. The absence of soft modes throughout the entire Brillouin zone suggests that no structural phase transition occurs at zero temperature, and the phonon spectra further support the thermodynamic stability of the investigated materials.

### 3.10. *Ab initio* molecular dynamics analysis

AIMD analysis serves as a powerful tool to bridge theoretical predictions and experimental conditions, ensuring that newly designed materials are not only stable in theory but also reliable in practical applications. Unlike static DFT calculations, which only provide ground-state properties, AIMD captures the dynamic evolution of a material's structure, energies, and thermodynamic parameters over time. This allows researchers to evaluate the thermal and dynamic stability of compounds, monitor fluctuations in total, kinetic, and potential energies, and track changes in temperature, pressure, and enthalpy, all of which are critical for determining whether a material can maintain structural integrity under realistic operating conditions.

Moreover, AIMD can reveal phase stability, anharmonic vibrations, melting behavior, lattice distortions, and resistance to external perturbations, providing insights into material performance beyond equilibrium.

**Fig. 9:**
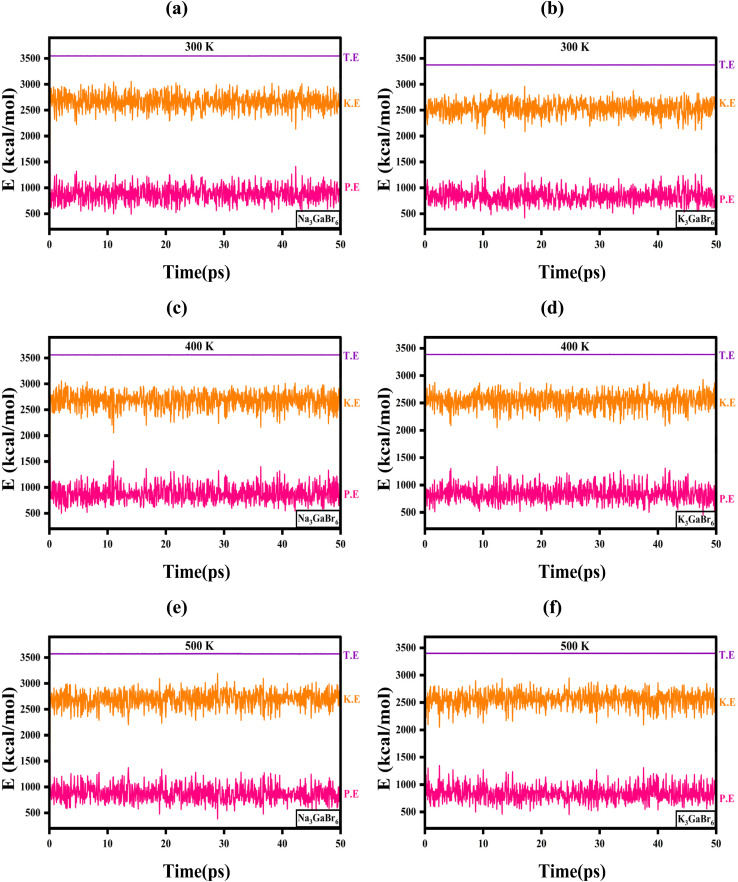
Time evolution of total (T.E.), kinetic (K.E.), and potential (P.E.) energies obtained from AIMD simulations for (a) Na_3_GaBr_6_ at 300 K, (b) K_3_GaBr_6_ at 300 K, (c) Na_3_GaBr_6_ at 400 K, (d) K_3_GaBr_6_ at 400 K, (e) Na_3_GaBr_6_ at 500 K, and (f) K_3_GaBr_6_ at 500 K.

The AIMD simulation results shown in Fig. 9(a–f) demonstrate the thermal and energetic stability of Na_3_GaBr_6_ and K_3_GaBr_6_ over a 50 ps time scale at 300 K, 400 K, and 500 K. For Na_3_GaBr_6_ (Fig. 9(a, c and e)), the total energy remains nearly constant at ∼3600 kcal mol^−1^ throughout the simulation at all temperatures, showing no systematic drift with time. The kinetic energy fluctuates around ∼2600 kcal mol^−1^ at 300 K, ∼2700 kcal mol^−1^ at 400 K, and ∼2850 kcal mol^−1^ at 500 K, consistent with the expected increase in atomic vibrations with temperature. Meanwhile, the potential energy stabilizes around ∼900–1100 kcal mol^−1^ with small oscillations, indicating that the crystal framework retains its structural integrity without any bond breaking or structural distortion. Similarly, for K_3_GaBr_6_ (Fig. 9(b, d and f)), the total energy remains stable at ∼3400 kcal mol^−1^ across the entire simulation period. The kinetic energy varies from ∼2500 kcal mol^−1^ at 300 K to ∼2700 kcal mol^−1^ at 500 K, while the potential energy fluctuates near ∼850–1000 kcal mol^−1^. The absence of any abrupt energy variation or drift confirms dynamic stability at elevated temperatures. The slightly lower total and potential energy values of K_3_GaBr_6_ compared to Na_3_GaBr_6_ suggest comparatively weaker lattice interactions and reduced vibrational stiffness, which is consistent with its softer lattice behavior observed in mechanical analysis. Overall, the steady energy profiles with temperature-dependent kinetic energy increase confirm that both compounds are thermally stable and dynamically robust up to 500 K.

**Fig. 10:**
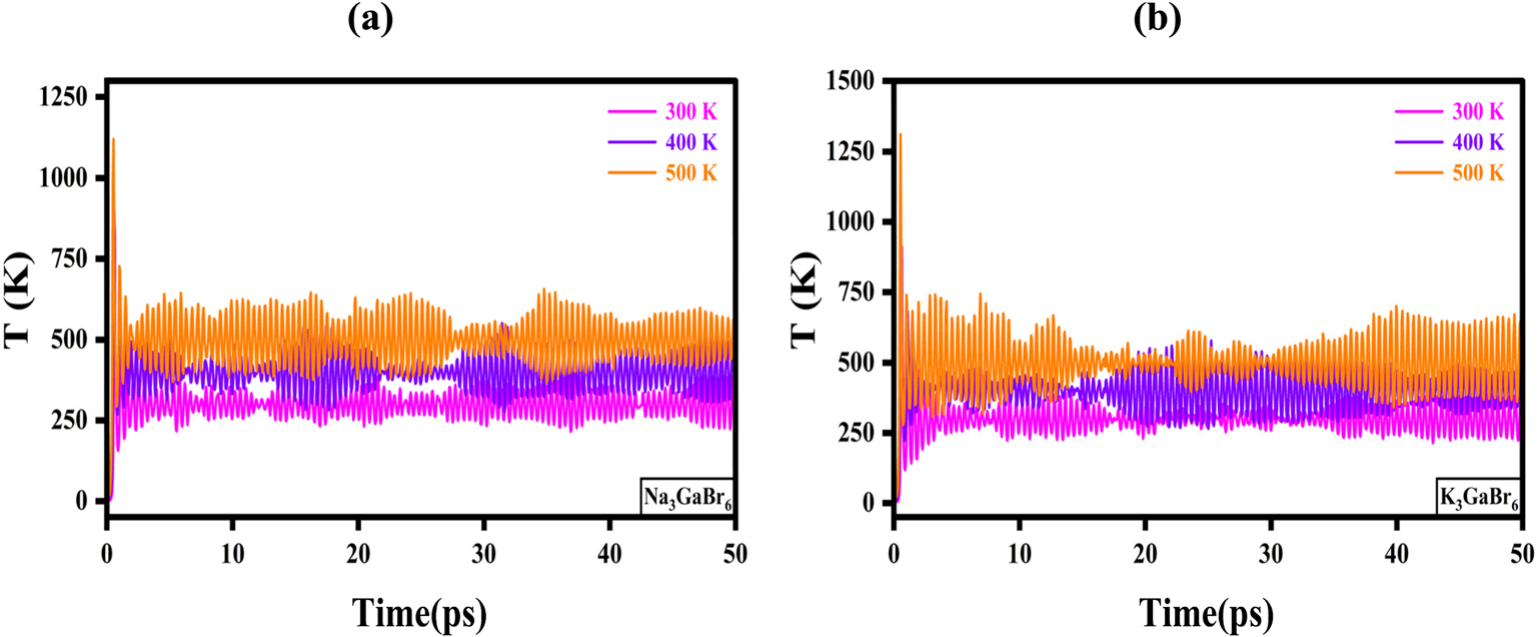
Temperature evolution during AIMD simulations of (a) Na_3_GaBr_6_ and (b) K_3_GaBr_6_ and at 300 K, 400 K, and 500 K.

The temperature evolution shown in Fig. 10 further confirms the thermal stability of both compounds during the 50 ps AIMD simulations. For Na_3_GaBr_6_ (Fig. 10(a)), the temperature fluctuates around the set values after initial equilibration, with averages of 300 K (ranging 250–350 K), 400 K (ranging 350–450 K), and 500 K (ranging 450–600 K). The oscillations slightly increase with temperature, which is expected due to enhanced atomic vibrations at higher thermal energy. Similarly, in Fig. 10(b), K_3_GaBr_6_ maintains stable temperature profiles centered near the target temperatures. At 300 K, the temperature varies within 260–340 K; at 400 K, within 350–460 K; and at 500 K, within 430–650 K. No systematic drift or abnormal thermal spike is observed after the equilibration period, confirming proper thermostat regulation and thermodynamic stability.

A comparison between the two compounds indicates that K_3_GaBr_6_ exhibits comparatively smoother and slightly narrower temperature fluctuations than Na_3_GaBr_6_, especially at elevated temperatures. This observation is consistent with the energy stability trends in Fig. 9 and suggests that K substitution contributes to improved dynamic and thermal stability of the lattice.

### 3.11. Experimental feasibility, chemical stability, and synthesis prospects

From an experimental viewpoint, the predicted negative formation energies, favorable tolerance factors (0.918 to 0.987), absence of imaginary phonon modes, and thermal robustness observed from AIMD simulations strongly indicate that Q_3_GaBr_6_ (Q = Na, K) compounds are chemically and structurally feasible for synthesis under laboratory conditions. The rigid GaBr_6_ octahedral framework and strong Ga–Br bonding suggests good resistance against lattice decomposition, while the alkali-metal cations provide ionic stabilization without participating directly in electronic degradation pathways. Similar vacancy-ordered A_3_BX_6_ halides such as Cs_3_InX_6_, Cs_3_SbX_6_, and Ag_3_BiI_6_ have already been experimentally synthesized using conventional solution processing, solid-state reactions, and flux growth methods, which indicates that Q_3_GaBr_6_ compounds are likely to be experimentally accessible through comparable synthesis routes. Possible fabrication strategies include solution-based crystallization using metal halide precursors, solid-state reactions under controlled atmospheres, and low-temperature melt or flux-assisted growth. However, challenges may arise from moisture sensitivity of bromide compounds and potential phase purity control, which require careful optimization of reaction temperature, stoichiometry, and encapsulation.

### 3.12. Population analysis

To further elucidate the electronic structure and bonding environment in the Q_3_GaBr_6_ (Q = Na, K) perovskites, a comprehensive Mulliken and Hirshfeld charge population analysis was carried out (Table 5). The low charge spilling values (0.13% for Na_3_GaBr_6_ and 0.10% for K_3_GaBr_6_) indicate reliable basis set convergence. In both compounds, the Q-site cations (Na^+^/K^+^) show partial charge transfer, with Mulliken charges of +0.50 (Na_1,2_) and +0.56 (K_1,2_), suggesting slight covalent interactions with nearby Br atoms, deviating from a purely ionic model. Notably, the third A-site ion in each case (Na_3_ = +0.19, K_3_ = −0.10) shows reduced or even reversed charge transfer, likely due to asymmetric local environments or polarization effects.

**Table 5:** Mulliken and Hirshfeld charge analysis of different atoms of Q_3_GaBr_6_ (Q = Na, K) perovskites

Compound	Change spilling	Species	Mulliken atomic populations					Mulliken change	Hirshfeld change
			s	P	d	f	Total		
Na_3_GaBr_6_	0.13%	Na (1)	2.14	6.37	0.00	0.00	8.50	0.50	0.36
		Na (2)	2.14	6.37	0.00	0.00	8.50	0.50	0.36
		Na (3)	2.24	6.57	0.00	0.00	8.81	0.19	0.15
		Ga	1.41	1.51	10.00	0.00	12.92	0.08	0.26
		Br	1.74	5.47	0.00	0.00	7.21	−0.21	−0.19
K_3_GaBr_6_	0.10%	K (1)	2.04	6.19	0.21	0.00	8.44	0.56	0.31
		K (2)	2.04	6.19	0.21	0.00	8.44	0.56	0.31
		K (3)	2.11	6.43	0.56	0.00	9.10	−0.10	0.17
		Ga	1.46	1.52	10.00	0.00	12.98	0.02	0.28
		Br	1.75	5.42	0.00	0.00	7.18	−0.18	−0.18

Ga atoms in both structures maintain almost neutral Mulliken charges (+0.08 in Na_3_GaBr_6_ and +0.02 in K_3_GaBr_6_), despite hosting significant d-electron density (∼10 e^−^), hinting at a delocalized bonding character with Br. The Br atoms, with negative Mulliken charges (−0.21 to −0.18), serve as primary electron acceptors, consistent with their higher electronegativity. Hirshfeld charge trends qualitatively support these observations but show slightly reduced magnitudes due to different partitioning criteria.^74,75^ The comparative results underscore the influence of ionic radius and electronegativity, Na^+^ exhibiting stronger localization than K^+^, and collectively suggest a complex interplay of ionic and covalent character in Ga–Br and A–Br bonds. These charge distributions align well with oxidation state expectations and provide deeper insight into the compounds' electrostatic stability and potential functional behavior.

## 4. Device performance and photovoltaic parameters

Driven by the favorable optoelectronic and structural properties of Q_3_GaBr_6_ (Q = Na, K), including suitable semiconducting behavior, mechanical robustness, strong light absorption, low optical loss, high refractive index, and confirmed thermodynamic and dynamical stability, we designed and simulated a Ni/Q_3_GaBr_6_/SnS_2_/FTO/Al perovskite solar cell using the SCAPS-1D platform. In this architecture, Q_3_GaBr_6_ serves as the absorber layer, while SnS_2_ is employed as the electron transport layer (ETL) due to its inorganic nature, excellent resistance to moisture and heat, low cost, non-toxicity, and ease of fabrication.^76^

**Fig. 11:**
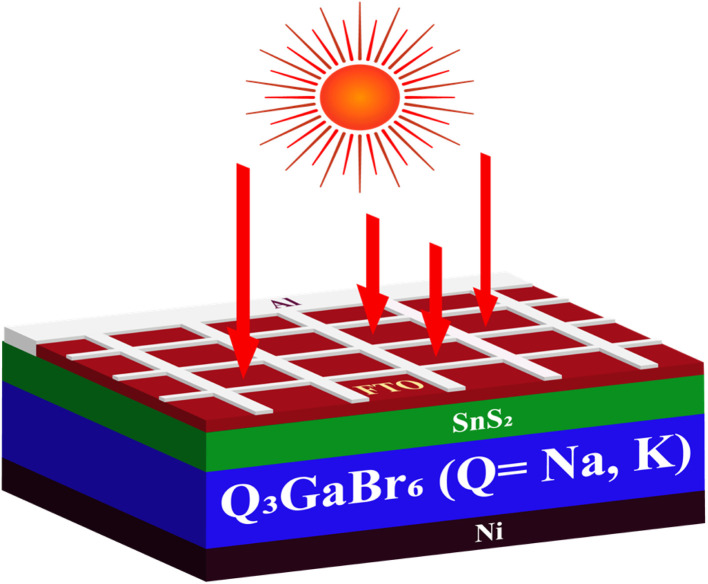
Schematic illustration of the device structure.

Nickel and aluminum are used as the back and front contacts,^77^ respectively, and fluorine-doped tin oxide (FTO) functions as the transparent conducting electrode.^78^ The schematic illustration of the device structure is presented in Fig. 11, and the material parameters utilized in the SCAPS-1D simulations are summarized in Table 6.

**Table 6:** Configurational parameters for the simulated device architecture

Parameters	FTO	SnS_2_	K_3_GaBr_6_	Na_3_GaBr_6_
Thickness (nm)	50	50	800	800
Band gap, *E*_g_ (eV)	3.6	2.24	1.445	1.991
Dielectric permittivity, *ε*_r_	10	10	3.7564	3.3865
Electron affinity, *χ* (eV)	4.5	4.24	3.655	3.109
*C*_B_ effective density of states, *N*_C_ (cm^−3^)	2 × 10^18^	2.2 × 10^18^	1.08 × 10^19^	1.240 × 10^19^
*V*_B_ effective density of states, *N*_V_ (cm^−3^)	1.8 × 10^19^	1.8 × 10^19^	1.476 × 10^19^	2.124 × 10^19^
Donor density, *N*_D_ (cm^−3^)	1 × 10^18^	1 × 10^17^	0	0
Acceptor density, *N*_A_ (cm^−3^)	0	0	1 × 10^17^	1 × 10^17^
Electron mobility, *µ*_n_ (cm^2^ V^−1^ s ^−1^)	50	50	90	80
Hole mobility, *µ*_h_ (cm^2^ V^−1^ s ^−1^)	20	50	38	44
Defect density, *N*_t_ (cm^−3^)	1 ×10^14^	1 × 10^14^	1 × 10^14^	1 × 10^14^

By evaluating parameters such as capture cross-section, defect type, and defect density, one can reveal how easily electrons or holes are trapped, whether the defects act as recombination centers, and how severely they limit the open-circuit voltage (*V*_OC_), short circuit current density (*J*_SC_), and power conversion efficiency (PCE).^79^ This analysis provides insights into interface quality, potential recombination losses, and pathways for optimizing material selection or surface passivation, ultimately guiding the design of high-performance solar cells.^80^

Table 7 summarizes the interfacial defect parameters of the SnS_2_/Q_3_GaX_6_ solar cell systems, with particular emphasis on the SnS_2_/Na_3_GaBr_6_ and SnS_2_/K_3_GaBr_6_ interfaces. In this work, a capture cross-section of 1 × 10^−19^ cm^2^ for both electrons and holes and a neutral defect type were considered, following commonly adopted assumptions for well-passivated heterointerfaces in numerical simulations. Similar ranges of capture cross-section (10^−18^–10^−20^ cm^2^) and neutral interface states have been widely reported in previous SCAPS and DFT-assisted device studies of chalcogenide/perovskite and metal-chalcogenide heterojunctions, where minimized trap activity is assumed to represent optimized interface conditions. The interfacial defect density was limited to 1 × 10^11^ cm^−2^, which is consistent with previously reported values for high-quality, chemically stable heterointerfaces in simulated perovskite and chalcogenide solar cells. Earlier studies have shown that interface defect densities in the range of 10^10^–10^12^ cm^−2^ correspond to well-passivated junctions with suppressed recombination losses, while higher values (>10^13^ cm^−2^) lead to severe carrier recombination and performance degradation.^81–83^ Therefore, the selected value represents a realistic yet optimistic scenario frequently adopted to model an upper-bound device performance under improved interface quality. These parameter choices indicate that the SnS_2_/Na_3_GaBr_6_ and SnS_2_/K_3_GaBr_6_ heterojunctions can be regarded as relatively clean and stable interfaces with low defect activity. Such conditions are highly favorable for efficient charge carrier transport across the junction and for minimizing non-radiative recombination losses in the simulated solar cells, while remaining consistent with defect density ranges reported in previous theoretical and simulation-based photovoltaic studies.

**Table 7:** Interfacial defect density of SnS_2_/Q_3_GaX_6_ (Q = Na and K) solar cells

Interfaces	Capture cross section: electrons/holes (cm^2^)	Defect type	Total defect density (cm^−2^)
SnS_2_/Na_3_GaBr_6_	1 × 10^−19^	Neutral	1 × 10^11^
SnS_2_/K_3_GaBr_6_	1 × 10^−19^	Neutral	1 × 10^11^

All simulations were carried out at a temperature of 300 K under an incident light intensity of 1000 W m^−2^, corresponding to the standard AM 1.5G solar illumination condition. To achieve optimal device performance, a comprehensive parametric optimization was conducted by systematically varying key absorber-layer properties, including the thickness, defect density, shallow acceptor concentration, and operating temperature. These parameters critically influence charge generation, recombination behavior, carrier transport, and overall device efficiency. Optimizing the absorber thickness ensures sufficient photon absorption while minimizing carrier recombination losses. Reducing defect density suppresses non-radiative recombination pathways and enhances carrier lifetime, while tuning the shallow acceptor concentration improves electrical conductivity and charge extraction efficiency. Additionally, the temperature dependence was analyzed to evaluate thermal stability and device reliability under realistic operating conditions. This optimization strategy provides a more reliable assessment of device performance and highlights the potential of the proposed solar cell structure for practical applications.

### 4.1. Energy band alignment analysis

**Fig. 12:**
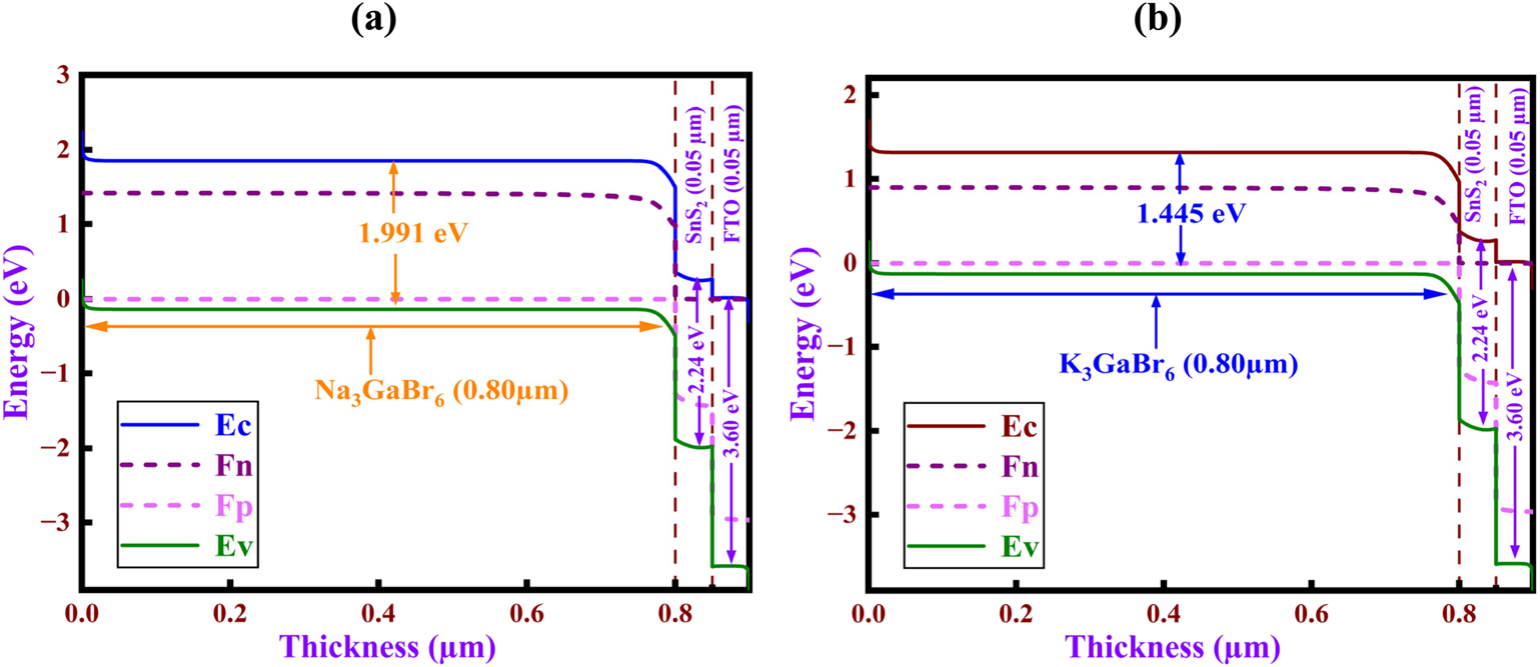
Energy band diagram of (a) Na_3_GaBr_6_ (b) K_3_GaBr_6_ materials.

Fig. 12(a) and (b) illustrate the energy band alignment of the Ni/Q_3_GaBr_6_/SnS_2_/FTO/Al solar cell, showing the variations of the conduction band (*E*_C_), valence band (*E*_V_), and quasi-Fermi levels for electrons (*F*_n_) and holes (*F*_p_) across the device thickness. For Na_3_GaBr_6_ (Fig. 12(a)) exhibits a wider bandgap of 1.991 eV, which may reduce absorption but favor a higher open-circuit voltage. In contrast K_3_GaBr_6_ (Fig. 12(b)), the bandgap is 1.445 eV, enabling strong visible-light absorption and efficient electron–hole generation. K_3_GaBr_6_ (Fig. 12(b)), the bandgap is 1.445 eV, enabling strong visible-light absorption and efficient electron–hole generation. In both devices, *F*_n_ stays close to *E*_C_ while *F*_P_ lies near *E*_v_ within the absorber, indicating effective charge separation and low bulk recombination. A favorable conduction band offset at the absorber/SnS_2_ interface facilitates electron transfer into the ETL while suppressing interfacial recombination. Since no hole transport layer is used, holes are collected directly by the Ni back contact, where the valence band alignment suggests near-ohmic behavior and effective electron blocking.

On the front side, electrons are transported through SnS_2_ to the FTO/Al contact with minimal energetic barriers, enabling efficient charge extraction. Overall, the band profiles indicate well-matched energy levels, enhanced carrier selectivity, and reduced recombination losses. K_3_GaBr_6_ is more suitable for light harvesting, whereas Na_3_GaBr_6_ offers potential for higher voltage output due to its wider bandgap, highlighting the complementary photovoltaic behavior of the two absorber materials.

### 4.2. Effect of absorber thickness on photovoltaic performance

**Fig. 13:**
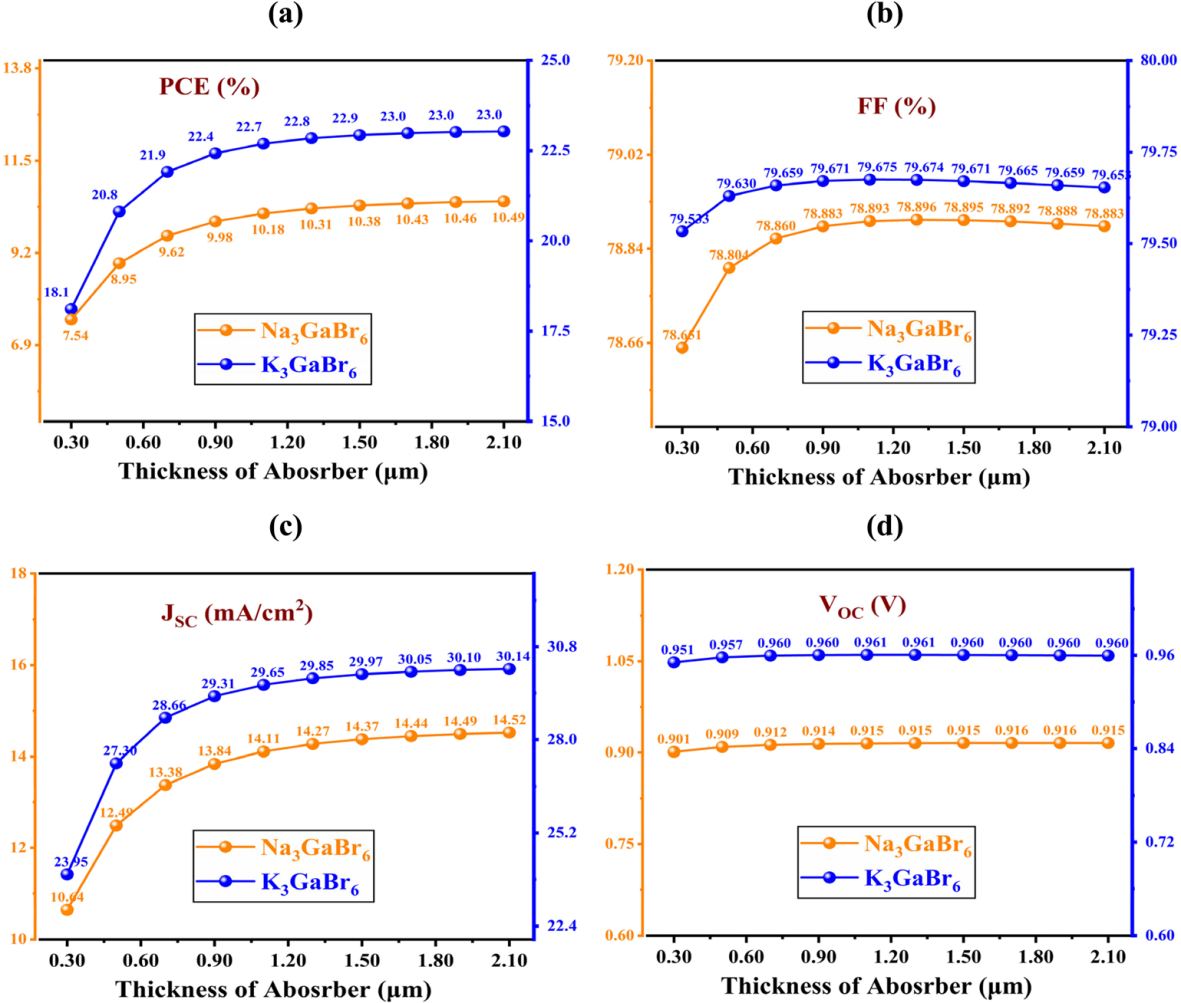
Variation of (a) PCE, (b) FF, (c) *J*_SC_, and (d) *V*_OC_ with absorber layer thickness for Na_3_GaBr_6_ and K_3_GaBr_6_-based solar cells.

Fig. 13 illustrates the variation of the photovoltaic performance parameters as a function of absorber layer thickness for the Q_3_GaBr_6_-based solar cell devices over a thickness range of 0.3 to 2.1 µm. A clear improvement in PCE, FF, and *J*_SC_ is observed for both materials as the absorber thickness increases, followed by saturation at higher thickness values.^84^ For Na_3_GaBr_6_, as shown in Fig. 13(a–c), the PCE, FF, and *J*_SC_ increase significantly from 7.54%, 78.651%, and 10.64 mA cm^−2^ at 0.3 µm to 9.83%, 78.87%, and 13.64 mA cm^−2^ at 0.8 µm, respectively, establishing 0.8 µm as the optimum thickness.

This improvement occurs because a thicker absorber enhances photon absorption, generating more charge carriers while still allowing efficient carrier collection. Beyond 0.8 µm, the improvement slows, and the values approach saturation at 10.49%, 78.883%, and 14.52 mA cm^−2^ at 2.1 µm. This behavior indicates that further thickness increase leads to longer carrier transport paths, which enhances recombination probability and reduces the effectiveness of additional light absorption.^85^ Similarly, for K_3_GaBr_6_, the performance metrics increase rapidly from 18.1%, 79.553%, and 23.95 mA cm^−2^ at 0.3 µm to 22.21%, 79.67%, and 29.04 mA cm^−2^ at 0.8 µm, confirming 0.8 µm as the optimal absorber thickness. With further increase in thickness, PCE, FF, and *J*_SC_ exhibit minor enhancement and finally saturate at 23.0%, 79.65%, and 30.14 mA cm^−2^ at 2.1 µm. As shown in Fig. 13(d), the *V*_OC_ remains nearly unchanged with increasing thickness, maintaining values of approximately 0.901 V for Na_3_GaBr_6_ and 0.951 V for K_3_GaBr_6_, which indicates that the absorber thickness has negligible influence on the device *V*_OC_. Notably, K_3_GaBr_6_ consistently exhibits superior performance across the entire thickness range, delivering more than twice the PCE and nearly double the *J*_SC_ compared to Na_3_GaBr_6_, while the FF values remain comparable (79%), indicating similar resistive losses. Bring it all together, K_3_GaBr_6_ emerges as the superior absorber material owing to its higher efficiency, stronger photocurrent, and improved voltage output, while 0.8 µm is identified as the optimum thickness for both absorber layers.

### 4.3. Effect of total defect density on photovoltaic performance

**Fig. 14:**
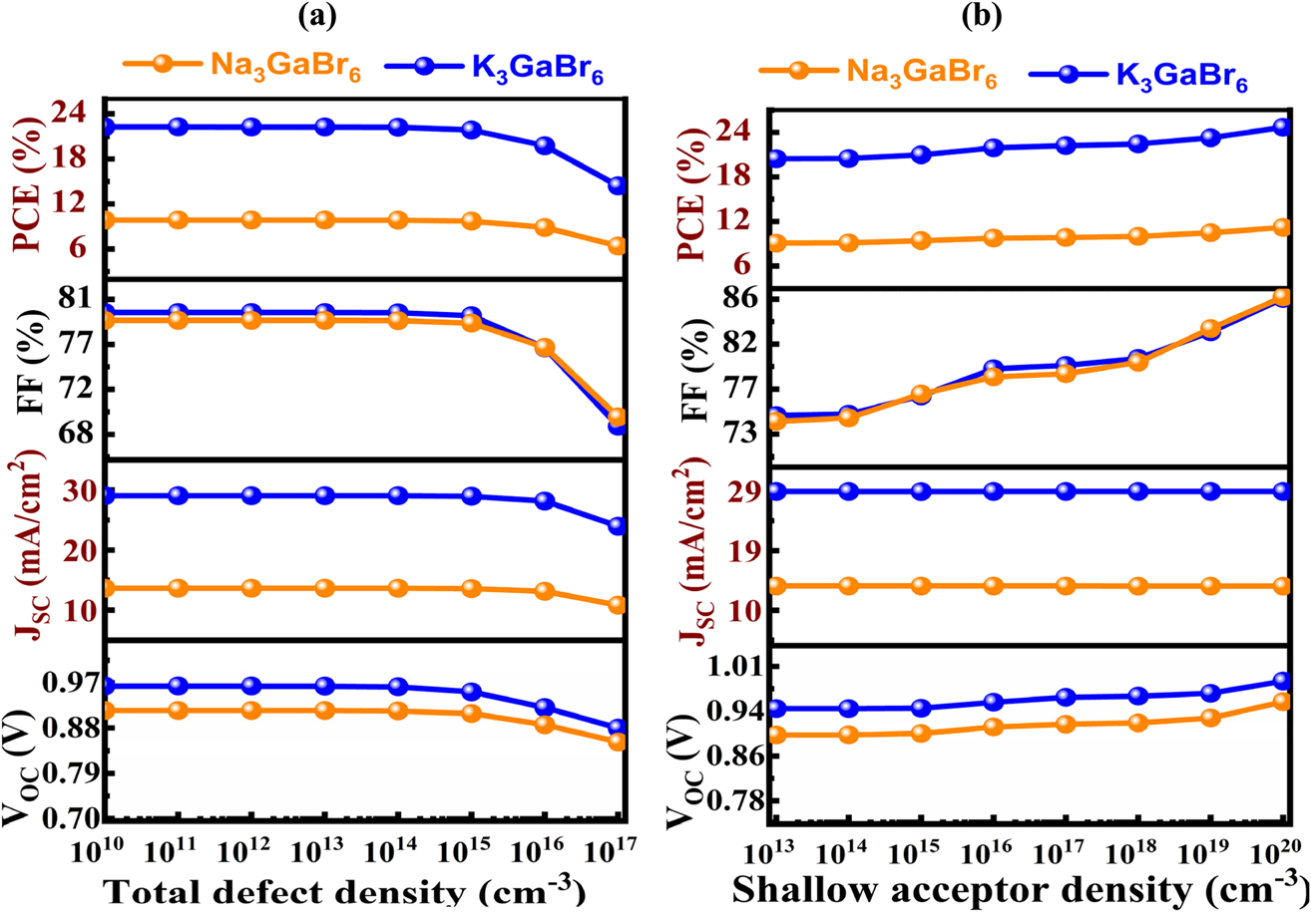
Variation of photovoltaic parameters of Na_3_GaBr_6_ and K_3_GaBr_6_ as a function of (a) total defect density and (b) shallow acceptor density.

Fig. 14(a) illustrates the dependence of photovoltaic performance parameters PCE, FF, *J*_SC_ and *V*_OC_ on the total defect density (*N*_t_) for Q_3_GaBr_6_ -based solar cell devices over a wide defect density range from 10^10^ to 10^17^ cm^−3^. At low and moderate *N*_t_ (10^10^ to 10^14^ cm^−3^), both devices exhibit nearly stable photovoltaic behavior. Within this range, the PCE remains almost constant at 9.83% for Na_3_GaBr_6_ and 22.21% for K_3_GaBr_6_, indicating strong tolerance against bulk defects at low concentrations. Notably, the K_3_GaBr_6_-based device consistently delivers superior performance compared to its Na_3_GaBr_6_ counterpart. This enhancement is mainly attributed to its significantly higher *J*_SC_, measured as 13.643 mA cm^−2^ for Na_3_GaBr_6_ and 29.041 mA cm^−2^ for K_3_GaBr_6_, along with its improved *V*_OC_ of 0.913 V for Na_3_GaBr_6_ and 0.960 V for K_3_GaBr_6,_ as shown in Fig. 14(a). These findings suggest more favorable charge transport characteristics and reduced recombination losses in the K_3_GaBr_6_ absorber layer, leading to improved photovoltaic performance under low-defect conditions.

When the *N*_t_ exceeds 10^14^ cm^−3^, both devices exhibit pronounced performance degradation, with the PCE decreasing to 6.391% for Na_3_GaBr_6_ and 14.41% for K_3_GaBr_6_ at 10^17^ cm^−3^. This decline is driven by the simultaneous reduction in *J*_SC_, FF, and *V*_OC_ due to the formation of deep-level defect states that enhance non-radiative recombination. The FF decreases from stable values of 78.87% (Na_3_GaBr_6_) and 79.66% (K_3_GaBr_6_) to 69.25% and 68.33%, respectively, while *J*_SC_ drops to 10.818 mA cm^−2^ and 23.96 mA cm^−2^ at 10^17^ cm^−3^ due to intensified Shockley–Read–Hall recombination.^86^ Likewise, *V*_OC_ declines to 0.853 V for Na_3_GaBr_6_ and 0.879 V for K_3_GaBr_6_, reflecting the increased recombination current at high defect densities.

In addition to defect-induced limitations, the lower efficiency of Na_3_GaBr_6_ is fundamentally constrained by the Shockley–Queisser limit associated with its wider bandgap, which restricts photon absorption and current generation even under ideal conditions.^87^ Consequently, both intrinsic thermodynamic losses and enhanced defect sensitivity contribute to the inferior performance of Na_3_GaBr_6_ compared to K_3_GaBr_6_. K_3_GaBr_6_, by contrast, exhibits superior defect tolerance and operates closer to its theoretical efficiency limit, highlighting its greater potential for high-performance photovoltaic applications.

### 4.4. Effect of shallow acceptor density

Fig. 14(b) illustrates the variation of photovoltaic parameters as a function of shallow acceptor density (*N*_A_) for Q_3_GaBr_6_ absorbers over the range of 10^13^ to 10^20^ cm^−3^. Increasing the shallow acceptor density enhances p-type conductivity by raising the hole concentration, which directly influences the absorber's built-in electric field, carrier transport properties, and recombination dynamics.^88^ At the initial doping level of 10^13^ cm^−3^, the PCE values are 9.07% for Na_3_GaBr_6_ and 20.45% for K_3_GaBr_6_. With the moderate *N*_A_ of 10^17^ cm^−3^, both materials exhibit notable improvement, achieving PCEs of 9.83% and 22.21%, which marks the most balanced operating point before the onset of diminishing returns at higher doping levels. At extreme doping (10^20^ cm^−3^), a further rise in PCE is observed, reaching 11.20% and 24.69%, though such high concentrations may not be practically favorable. A similar trend is observed for FF and *V*_OC_: from initial (10^13^ cm^−3^) values of 74.33% and 0.895 V for Na_3_GaBr_6_ and 74.88% and 0.941 V for K_3_GaBr_6_, both parameters increase at the moderate *N*_A_ = 10^17^ cm^−3^ to 78.87% and 0.913 V for Na_3_GaBr_6_, and 79.66% and 0.96 V for K_3_GaBr_6_, reflecting enhanced carrier extraction and reduced recombination. At 10^20^ cm^−3^, FF rises further to above 86.25% and 86.05 for both materials, while *V*_OC_ increases only modestly. Throughout the entire *N*_A_ ranges, *J*_SC_ remains nearly constant 13.64 mA cm^−2^ for Na_3_GaBr_6_ and 29.04 mA cm^−2^ for K_3_GaBr_6_ indicating that acceptor density has little effect on optical absorption or carrier generation.

### 4.5. Effect of temperature variation

The influence of temperature on the device performance of Na_3_GaBr_6_- and K_3_GaBr_6_-based solar cells is governed by several intrinsic physical mechanisms, including bandgap shrinkage, enhanced carrier-phonon scattering, increased intrinsic carrier concentration, and temperature-activated recombination. As the operating temperature rises, lattice vibrations intensify, leading to reduced carrier mobility and a higher probability of recombination through both radiative and non-radiative pathways. Additionally, temperature-induced bandgap narrowing decreases *V*_OC_ due to its inverse dependence on the saturation current density. These combined effects result in the systematic degradation of photovoltaic performance at elevated temperatures.

**Fig. 15:**
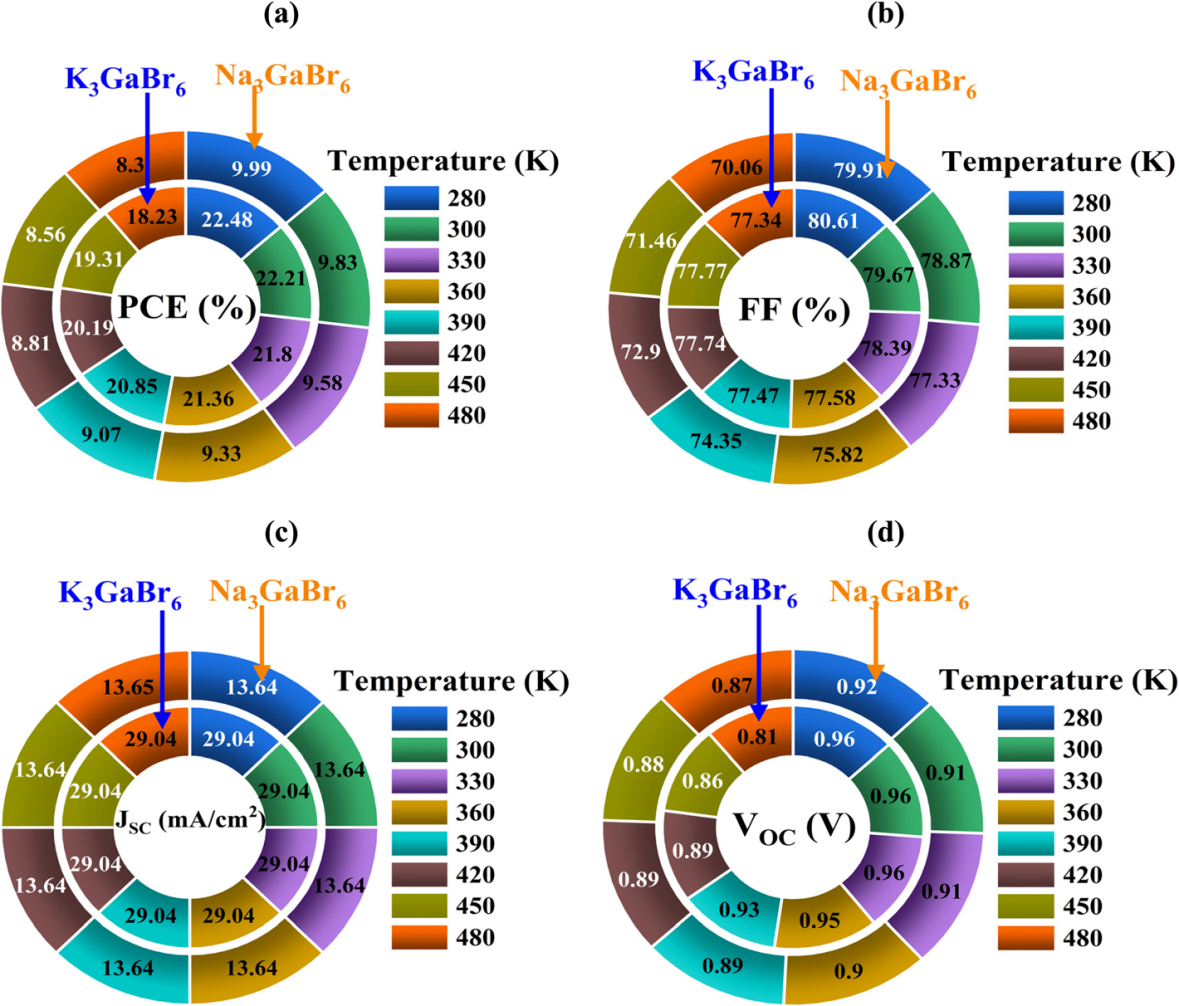
Temperature-dependent photovoltaic parameters ((a) PCE, (b) FF, (c) *J*_SC_, (d) *V*_OC_) of Na_3_GaBr_6_ and K_3_GaBr_6_-based solar cells (280 to 480 K).

In Fig. 15(a), this behavior is reflected in the temperature-dependent variation of PCE. At 280 K, the devices show efficiencies of 9.99% and 22.48% for Na_3_GaBr_6_ and K_3_GaBr_6_, respectively. When the temperature is increased to 300 K, the PCE slightly decreases to 9.83% and 22.21%, indicating the onset of thermally enhanced recombination and minor mobility loss. A further increase to 390 K causes more pronounced degradation, with PCE dropping to 9.07% for Na_3_GaBr_6_ and 20.85% for K_3_GaBr_6_. At 480 K, this trend continues, and the efficiencies fall to 8.30% and 18.23%, respectively.

These reductions are consistent with theoretical expectations, as higher temperatures accelerate recombination and weaken the built-in potential essential for efficient carrier extraction. A similar temperature dependence is observed in Fig. 15(b) and (d) for FF and *V*_OC_. At 280 K, Na_3_GaBr_6_ exhibits an FF of 79.91% and *V*_OC_ of 0.92 V, while K_3_GaBr_6_ shows 80.61% and 0.96 V. Increasing the temperature to 300 K slightly reduces the FF to 78.87% and 79.67%, but *V*_OC_ remains nearly unchanged at 0.91 V and 0.92 V due to the relatively small bandgap shift in this temperature window. However, at 480 K, the combined effects of increased recombination and reduced carrier mobility significantly degrade both parameters, with FF and *V*_OC_ decreasing to 70.06% and 0.87 V for Na_3_GaBr_6_ and 77.34% and 0.81 V for K_3_GaBr_6_. In contrast, Fig. 15(c) shows that *J*_SC_ remains effectively constant over the entire temperature range, with values of 13.64 mA cm^−2^ for Na_3_GaBr_6_ and 29.04 mA cm^−2^ for K_3_GaBr_6_. This nearly unchanged behavior indicates that the optical absorption coefficient and photogenerated carrier density are minimally affected by temperature. Since *J*_SC_ primarily depends on photon flux and absorption rather than on carrier recombination mechanisms, its stability is consistent with theoretical predictions. Overall, the combined theoretical framework and simulation results clearly demonstrate that while temperature variations significantly influence *V*_OC_, FF, and PCE due to enhanced recombination and reduced mobility, *J*_SC_ remains unaffected. Despite the observed degradation at high temperatures, the Q_3_GaBr_6_ absorbers retain a reasonably stable performance profile, confirming their potential suitability for environments with moderate thermal fluctuations.

### 4.6. *JV* and QE

**Fig. 16:**
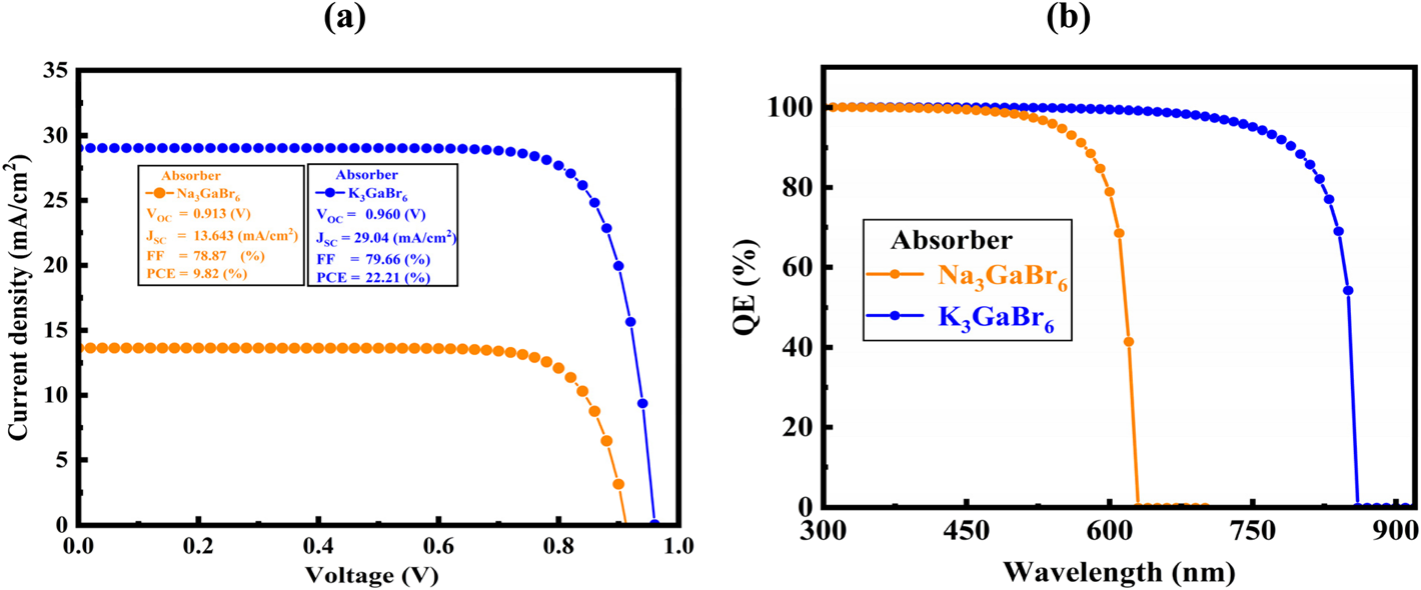
(a) *J*–*V* curves and (b) QE spectra of Na_3_GaBr_6_ and K_3_GaBr_6_ absorber-based solar cells.

The *J*–*V* characteristics presented in Fig. 16(a) clearly distinguish the photovoltaic behavior of the Na_3_GaBr_6_- and K_3_GaBr_6_-based solar cells, with the K_3_GaBr_6_ device demonstrating markedly superior performance. The higher *V*_OC_ of 0.960 V, enhanced *J*_SC_ of 29.04 mA cm^−2^, and elevated PCE of 22.21% obtained for K_3_GaBr_6_ indicate more efficient charge generation and reduced recombination losses within the absorber layer. In contrast, the Na_3_GaBr_6_ device exhibits comparatively lower *V*_OC_ of 0.913 V and *J*_SC_ of 13.643 mA cm^−2^, resulting in a reduced PCE of 9.82%, primarily due to its limited current-producing capability.

These differences are strongly supported by the quantum efficiency spectra shown in Fig. 16(b), where K_3_GaBr_6_ maintains a high QE exceeding 90% over a broad wavelength range and extends its spectral response up to approximately 900 nm. Conversely, Na_3_GaBr_6_ displays a significantly narrower absorption window, with QE rapidly declining beyond 600 nm, consistent with its larger effective bandgap and weaker long-wavelength absorption. The combined *J*–*V* and QE analyses confirm that K_3_GaBr_6_ possesses superior optoelectronic properties such as improved light-harvesting ability, longer carrier diffusion lengths, and reduced recombination which collectively contribute to its enhanced solar cell performance compared with Na_3_GaBr_6_.

### 4.7. Overall device performance

**Fig. 17:**
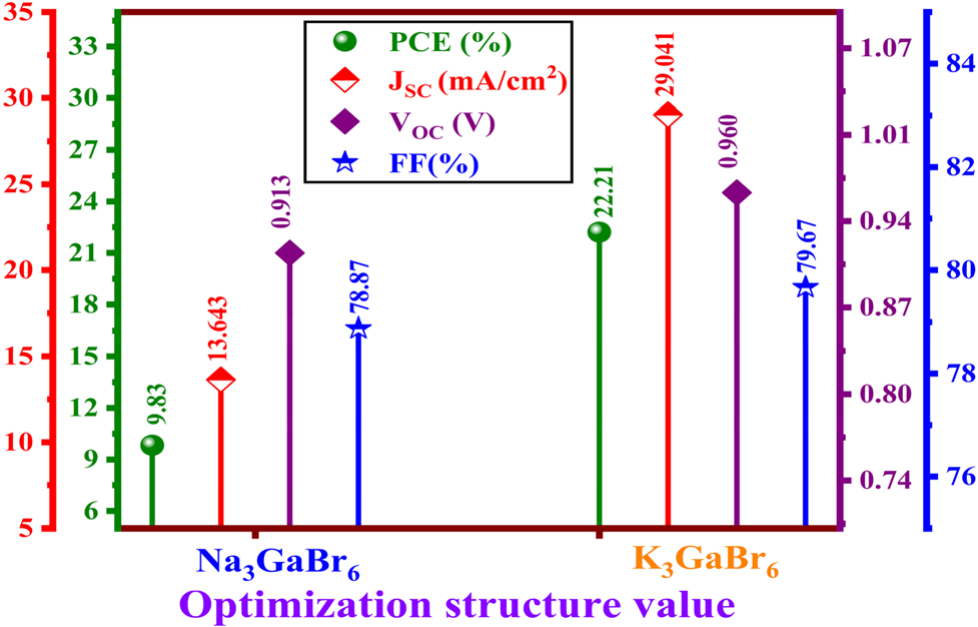
Comparative photovoltaic performance parameters PCE, *J*_SC_, *V*_OC_, and FF of optimized Na_3_GaBr_6_ and K_3_GaBr_6_ perovskite structures.

Under the optimized device conditions comprising an absorber thickness of 0.8 µm, shallow acceptor density of 10^17^ cm^−3^, total defect density of 10^14^ cm^−3^, an operating temperature of 300 K, and an interface defect density of 10^11^ cm^−2^ the Q_3_GaBr_6_ (Q = Na, K) double perovskite absorbers exhibit distinct photovoltaic performances. In Fig. 17, a clear comparison between Na_3_GaBr_6_ and K_3_GaBr_6_ shows that the K-based material significantly surpasses its Na counterpart. The Na_3_GaBr_6_ device achieves a PCE of 9.83%, with a *J*_SC_ of 13.643 mA cm^−2^, *V*_OC_ of 0.913 V, and FF of 78.87%.

Under the same idealized simulation conditions, K_3_GaBr_6_ delivers markedly enhanced performance, reaching a PCE of 22.21%, with a much higher *J*_SC_ of 29.041 mA cm^−2^, *V*_OC_ of 0.960 V, and a FF of 79.67%. This improvement can be attributed to enhanced photon absorption, more favorable charge transport, and reduced recombination losses predicted for the K-based compound. It is important to emphasize that these results represent theoretical upper-limit device performances obtained from SCAPS-1D simulations under optimized and ideal assumptions. Therefore, they should not be interpreted as realistic experimental efficiencies but rather as an indication of the intrinsic photovoltaic potential of these materials. To the best of our knowledge, prior studies have not reported photovoltaic performance simulations for A_3_BX_6_-type materials, and this work provides initial simulation-based insight into their possible device behavior and efficiency limits.

## 5. Potential applications of Q_3_GaBr_6_ (Q = Na, K)

Based on the comprehensive first-principles and device-level investigations, the vacancy-ordered halide perovskites Q_3_GaBr_6_ (Q = Na, K) exhibit a unique combination of suitable band gaps, strong optical absorption in the visible and ultraviolet regions, good mechanical stability, and excellent thermal and dynamical robustness. These characteristics make them promising candidates for several optoelectronic and energy-related applications. The direct band gaps of 1.445 to 1.991 eV (GGA-PBE) and their corrected values from mGGA-rSCAN and HSE06 place Q_3_GaBr_6_ within the optimal range for photovoltaic absorbers, enabling efficient sunlight harvesting and charge-carrier generation. Their high absorption coefficients (>10^4^ cm^−1^ in the visible region) and low reflectivity further enhance their suitability as absorber layers in lead-free perovskite solar cells. Moreover, the strong ultraviolet absorption and pronounced dielectric response suggest potential applications in UV photodetectors, optical sensors, and radiation-detection devices. The moderate refractive index and low optical losses in the visible region also indicate possible use in transparent optoelectronic coatings and antireflection layers. From a mechanical and thermal standpoint, the dynamical stability confirmed by phonon dispersion and the excellent thermal robustness observed in AIMD simulations imply that these materials can withstand realistic device-operating conditions, making them viable for long-term practical deployment. In particular, the ductile nature and higher mechanical flexibility of K_3_GaBr_6_ make it especially attractive for thin-film and flexible optoelectronic devices. Finally, the successful integration of DFT-derived parameters into SCAPS-1D simulations demonstrates that Q_3_GaBr_6_ compounds are not only theoretically stable but also technologically relevant, bridging the gap between material discovery and device engineering. Therefore, Na_3_GaBr_6_ and especially K_3_GaBr_6_ emerge as promising lead-free absorber materials for next-generation solar cells, UV photodetectors, and other sustainable optoelectronic applications.

## 6. Limitation

This study is subject to several theoretical limitations arising from both the DFT calculations and the device-level simulations. From the DFT perspective, the calculated electronic and optical properties do not explicitly account for excitonic effects, which can be significant in halide perovskites and may influence light absorption and carrier dynamics. Although advanced functionals (HSE06 and mGGA-RSCAN) were employed to improve band gap accuracy, inherent approximations of DFT still remain. From the device simulation perspective, the SCAPS-1D results were obtained under idealized assumptions. Key parameters such as defect density, carrier mobility, and recombination velocities were either optimized within the simulator or assumed within reasonable ranges due to the absence of experimental data for these materials. Therefore, the predicted photovoltaic efficiencies should be regarded as theoretical upper-limit estimates rather than realistic device performance. In practical implementations, material imperfections, interface losses, and additional physical effects not considered here may lead to reduced efficiencies.

## 7. Conclusion

This work establishes an efficient multiscale framework by coupling first-principles calculations with SCAPS-1D device modeling, enabling a direct and systematic link between intrinsic material properties and photovoltaic performance. Such an integrated strategy minimizes computational overhead and experimental uncertainty, providing a practical pathway for the rapid identification and optimization of promising lead-free absorber materials. Moreover, we report the first complete theoretical and device-level assessment of the vacancy-ordered halide perovskites Q_3_GaBr_6_ (Q = Na, K) for photovoltaic and optoelectronic applications. The DFT results verify that Na_3_GaBr_6_ and K_3_GaBr_6_ adopt a cubic *Fm*3̄*m* structure with thermodynamically stable configurations, supported by negative formation energies, suitable tolerance factors, and structurally rigid GaBr_6_ octahedra. These characteristics confirm their structural robustness and highlight their potential as stable, environmentally benign alternatives to conventional Pb-based perovskite absorbers. Their direct band gaps, strong dielectric response, and high absorption coefficients (>10^4^ cm^−1^ in the visible range) highlight their potential as efficient light-harvesting materials. Mechanical and phonon analyses, along with AIMD simulations, further demonstrate that both compounds possess excellent thermodynamic and dynamical stability in different temperature, with K_3_GaBr_6_ exhibiting slightly enhanced structural robustness and ductility compared to Na_3_GaBr_6_. Device-level simulations using SCAPS-1D, incorporating DFT-derived parameters, reveal that absorber thickness, defect concentration, and acceptor density significantly influence photovoltaic performance. Both materials show promising behavior under optimized conditions, indicating their viability as Pb-free absorber layers. At the optimized absorber thickness of 0.8 µm, the Na_3_GaBr_6_-based device achieves a PCE of 9.83%, whereas the K_3_GaBr_6_-based device reaches a significantly higher theoretical upper-limit PCE of 22.21%, owing to its superior *J*_SC_ (29.04 mA cm^−2^) and *V*_OC_ (0.96 V).

## Ethical statement

The manuscript's authors agree that there is no research involving human participants, human data or tissue, or animal subjects.

## Author contributions

Rifat Rafiu, Md. Sakib Hasan: methodology, validation, software, conceptualization, investigation, formal analysis, data curation, visualization, writing – original draft, and review and editing. Md. Azizur Rahman, Imtiaz Ahamed Apon, Karim Kriaa, Mohamed Benghanem, S. AlFaify, Noureddine Elboughdiri: investigation, validation, software, formal analysis, data curation, writing – original draft, and review and editing.

## Conflicts of interest

The authors have no conflicts of interest.

## Data Availability

Data will be made available on reasonable request.
